# Gut-resident microorganisms and their genes are associated with cognition and neuroanatomy in children

**DOI:** 10.1126/sciadv.adi0497

**Published:** 2023-12-22

**Authors:** Kevin S. Bonham, Guilherme Fahur Bottino, Shelley Hoeft McCann, Jennifer Beauchemin, Elizabeth Weisse, Fatoumata Barry, Rosa Cano Lorente, The RESONANCE Consortium, Curtis Huttenhower, Muriel Bruchhage, Viren D’Sa, Sean Deoni, Vanja Klepac-Ceraj

**Affiliations:** ^1^ Department of Biological Sciences, Wellesley College, Wellesley, MA, USA.; ^2^ Rhode Island Hospital, Providence, RI, USA.; ^3^ Department of Psychology, University of Stavanger, Stavanger, Norway.; ^4^ Department of Biostatistics, Harvard T.H. Chan School of Public Health, Boston, MA, USA.; ^5^ Department of Immunology and Infectious Diseases, Harvard T.H. Chan School of Public Health, Boston, MA, USA.; ^6^ Harvard Chan Microbiome in Public Health Center, Harvard T.H. Chan School of Public Health, Boston, MA, USA.; ^7^ Associate Member, Broad Institute of MIT and Harvard, Cambridge, MA, USA.

## Abstract

Emerging evidence implicates gut microbial metabolism in neurodevelopmental disorders, but its influence on typical neurodevelopment has not been explored in detail. We investigated the relationship between the microbiome and neuroanatomy and cognition of 381 healthy children, demonstrating that differences in microbial taxa and genes are associated with overall cognitive function and the size of brain regions. Using a combination of statistical and machine learning models, we showed that species including *Alistipes obesi*, *Blautia wexlerae*, and *Ruminococcus gnavus* were enriched or depleted in children with higher cognitive function scores. Microbial metabolism of short-chain fatty acids was also associated with cognitive function. In addition, machine models were able to predict the volume of brain regions from microbial profiles, and taxa that were important in predicting cognitive function were also important for predicting individual brain regions and specific subscales of cognitive function. These findings provide potential biomarkers of neurocognitive development and may enable development of targets for early detection and intervention.

## Teaser

The complex community of microorganisms living in the guts of infants and children may affect how their brains develop.

## INTRODUCTION

The gut and the brain are intimately linked. Signals from the brain reach the gut through the autonomic nervous system and the endocrine system, and the gut can communicate with the brain through the vagus nerve and through endocrine and immune (cytokine) signaling molecules (*1*–*4*). In addition, the products of microbial metabolism generated in the gut can influence the brain, both indirectly by stimulating the enteric nervous and immune systems and directly through molecules that enter circulation and cross the blood-brain barrier. Causal links between the gut microbiome and neural development, particularly atypical development, are increasingly being identified (*5*). Both human epidemiology and animal models point to the effects of gut microbes on the development of autism spectrum disorder (*6*, *7*), and specific microbial taxa have been associated with depression (*8*, *9*) and Alzheimer’s disease (*10*, *11*). However, information about this “microbiome-gut-brain axis” in normal neurocognitive development remains lacking, particularly early in life.

The first years of life are critical developmental windows for both the microbiome and the brain (*12*). Fetal development is believed to occur in a sterile environment, but newborns are rapidly seeded at birth through contact with the birth canal (if birthed vaginally), caregivers, food (breast milk or formula), and other environmental sources (*13*, *14*). The early microbiome is characterized by low microbial diversity, rapid succession, and evolution and is dominated by Actinobacteria, particularly the genus *Bifidobacterium*, Bacteroidetes, especially *Bacteroides*, and Proteobacteria (*15*). Many of these bacteria have specialized metabolic capabilities for digesting human milk, such as *Bifidobacterium longum* subsp. *infantis* and *Bacteroides fragilis* (*16*). Upon the introduction of solid foods, the gut microbiome undergoes another categorical transformation; its diversity increases, and most taxa of the infant microbiome are replaced by taxa more reminiscent of adult microbiomes (*13*). Prior studies have typically focused on either infant microbiomes or adult microbiomes, since performing statistical analyses across this transition poses particular challenges. Nevertheless, since this transition coincides with critical neural developmental windows and associated neurodevelopmental processes including myelination, neurogenesis, and synaptic pruning, investigation across this solid-food boundary is important (*17*).

A child’s brain undergoes remarkable anatomical, microstructural, organizational, and functional changes in the first years of life. By age 5, a child’s brain has reached >85% of its adult size and achieved near-adult levels of myelination, and the pattern of axonal connections has been established (*18*). Much of this development occurs in discrete windows called sensitive periods (SPs) (*19*) during which neural plasticity is particularly high. Emerging evidence suggests that the timing and duration of SPs may be driven in part by cues from the developing gut microbiome (*20*, *21*). As such, understanding the normal spectrum of healthy microbiome development and how it relates to normal neurocognitive development may provide opportunities for identifying atypical development earlier and offer opportunities for intervention.

To begin to address this need, we investigated relationship between the gut microbiome and neurocognitive development in a large cohort of healthy and neurotypically developing children from infancy through 10 years of age. Gut microbial communities were assessed using shotgun metagenomic sequencing, enabling profiling at both the taxonomic and gene-functional level. Cognitive skills and abilities were measured using age-appropriate psychometric assessments of cognitive function, i.e., the Mullen Scales of Early Learning (MSEL) (*22*), Wechsler Preschool and Primary Scale of Intelligence, 4th Edition (WPPSI-4) (*23*) and Wechsler Intelligence Scale for Children, 5th Edition (WISC-V) (*24*). Finally, we also assessed emerging brain structure using magnetic resonance imaging (MRI). Through a combination of classical statistical analysis and machine learning (ML), we find that the development of the gut microbiome, children’s cognitive abilities, and brain structure are intimately linked, with both microbial taxa and gene functions able to predict cognitive performance and brain structure.

## RESULTS

### The RESONANCE cohort is part of a study of child development

**Fig. 1.**
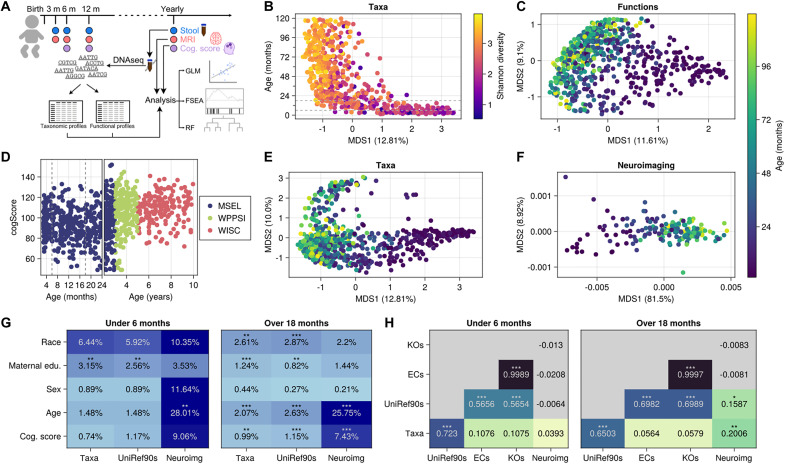
The RESONANCE cohort is a diverse cohort with accelerated longitudinal sampling. (**A**) Stool samples, cognitive assessments, and neuroimaging were collected from participants at different ages throughout the first years of life. (**B**) Cognitive function scores are assessed by different tests, but may be normalized using full-scale composite scores. (**C** and **D**) Principal coordinates analysis using Bray-Curtis dissimilarity on taxonomic profiles demonstrates high beta diversity, with much of the first axis of variation explained by increasing age and alpha diversity. MSEL, Mullen Scales of Early Learning; WPPSI, Wechsler Preschool & Primary Scale of Intelligence; WISC, Wechsler Intelligence Scale for Children. Differences in gene function profiles (**E**) and neuroimaging [principal components analysis (PCA) based on the Euclidean distance of brain region volumes] (**F**) are likewise dominated by changes as children age. (**G**) Permutation analysis of variance (PERMANOVA) of taxonomic profiles, functional profiles (annotated as UniRef90s), and neuroimaging against the metadata of interest. Variations in taxonomic and functional profiles explain a modest but significant percent of the variation in cognitive development in children over 18 months of age. (**H**) Mantel testing of different microbial feature matrices shows overlapping but distinct patterns of variation. Dotted lines in (B) and (C) show 6 and 18 months, which are used as cutoffs in some models.

We investigated the co-development of the brain and microbiome in 381 healthy and neurotypically developing children (172 female) between 40 days and 10 years of age (Table 1, Fig. 1A, and fig. S1) using a variety of orthogonal microbial and neurocognitive measures. These included shotgun metagenomic sequencing, age-appropriate cognitive and behavioral assessments using full-scale composite scores from MSEL, WPPSI-4, and WISC-V, and neuroimaging measures of cortical and subcortical morphology. Different assessment instruments were used depending on the child’s age due to the unique age range and psychometric properties measured by each (i.e., MSEL for children under 4 years of age, WPPSI-3 for 3 to 6 year olds, and WISC-V for children older than 6), but were normalized to a common scale (Fig. 1D), As expected, the greatest differences in microbial taxa were observed across child age (Fig. 1, B and E; principle coordinates analysis axis 1), with older children primarily stratified into Bacteroidetes-dominant, Firmicutes-dominant, or high-abundance *Prevotella copri* (fig. S2). Overall subject-to-subject variation in gut microbial genes and brain volume profiles was similarly driven largely by subject age (Fig. 1, C and F).

**Table 1.** Subjects in the ECHO RESONANCE cohort come from a diverse cross section of Rhode Island families. “All” refers to the entire cohort. “Under 6 months,” “Over 18 months,” and “Future” refer specifically to samples that were used included in the respective subgroup analyses.

Group	Subgroup	All	Under 6 months	Over 18 months	Future
*N* subjects		381	82	277	104
Samples	1	214 (56.2%)			
	2	90 (23.6%)			
	>2	77 (20.2%)			
Age (months)	Min	1.3	2.89	18.03	1.3
	Max	119.53	5.95	119.53	11.95
	Median	24.62	3.64	41.59	6.08
Sex	F	172 (45.1%)	42 (51.2%)	122 (44.0%)	47 (45.2%)
	M	209 (54.9%)	40 (48.8%)	155 (56.0%)	57 (54.8%)
Race	White	265 (69.6%)	48 (58.5%)	201 (72.6%)	62 (59.6%)
	Black	40 (10.5%)	15 (18.3%)	21 (7.6%)	17 (16.3%)
	Indigenous	1 (0.3%)	0 (0.0%)	1 (0.4%)	0 (0.0%)
	Asian	8 (2.1%)	2 (2.4%)	7 (2.5%)	3 (2.9%)
	Mixed	54 (14.2%)	14 (17.1%)	38 (13.7%)	16 (15.4%)
	Other	3 (0.8%)	1 (1.2%)	3 (1.1%)	2 (1.9%)
Maternal education	Junior high school	2 (0.5%)	0 (0.0%)	2 (0.7%)	0 (0.0%)
	Some high school	6 (1.6%)	3 (3.7%)	4 (1.4%)	2 (1.9%)
	High school grad	39 (10.2%)	13 (15.9%)	22 (7.9%)	17 (16.3%)
	Some college	100 (26.2%)	28 (34.1%)	60 (21.7%)	30 (28.8%)
	College grad	95 (24.9%)	19 (23.2%)	75 (27.1%)	30 (28.8%)
	Grad/professional school	126 (33.1%)	16 (19.5%)	106 (38.3%)	23 (22.1%)

As several previous studies have demonstrated associations between specific gut taxa and atypical neurocognition and neurological disorders (*7*, *8*, *25*–*27*), we sought to determine whether specific taxa or gene functions were associated with normal cognitive development in children. To test whether variations in gut microbial taxa, their genes, or their metabolism were associated with neurocognitive development, we used permutation analysis of variance (PERMANOVA) that included the β diversity of microbial taxa and gene functions, neuroimaging-derived volume measures of cortical and subcortical structures, and general cognitive ability. Given the large ecological shift in the microbiome that occurs upon the introduction of solid food, and the relatively wide range of ages when over which infant transition from milk to solid foods, we separately considered ages that are generally pre-transition (before 6 months of age) and those that are generally post-transition (over 18 months of age). However, even with this categorization, we find increasing variation in gut microbiomes in children over 18 months old (Fig. 1G). We also found that overall variation in microbial species in children over 18 months old was significantly associated with variation in cognitive function score (Fig. 1G; *R*^2^ = 0.0099, *q* < 0.01), as was variation in microbial gene functions (*R*^2^ = 0.0115, *q* < 0.001). Variation in microbial taxa and genes was not significantly associated with cognitive function in children under 6 months, although this may be due to the low taxonomic diversity and broad lack of overlap between taxa in infants (Fig. 1G). As expected, age was significantly associated with microbial beta diversity [taxa *R*^2^ = 0.0207, and gene functions annotated with UniRef90s clusters of 90% similarity (*28*): *R*^2^ = 0.0258, *q* < 0.001] and very strongly associated with variation in neuroimaging (MRI) profiles (*R*^2^ = 0.258, *q* < 0.001).

Consistent with previous studies, different microbial measurement types captured overlapping variation, with species profiles and gene function profiles, both generated from metagenomic sequences, being tightly coupled (Fig. 1H; *P* < 0.001). Other functional groupings [Enzyme commission level4 (ECs) (*29*) and Kyoto Encyclopedia of Genes and Genomes orthologs (KOs) (*30*)] overlapped only slightly with taxonomic profiles in both age cohorts, despite being derived from UniRef90 labels. In children over 18 months, some variation (taxa: 20.1%, *P* < 0.01; gene functions: 15.9%, *P* < 0.05) in neuroimaging overlapped with microbial measures, although this may be due to the residual variation because of age in both measures.

### Microbial species and neuroactive genes are associated with cognitive performance

**Fig. 2.**
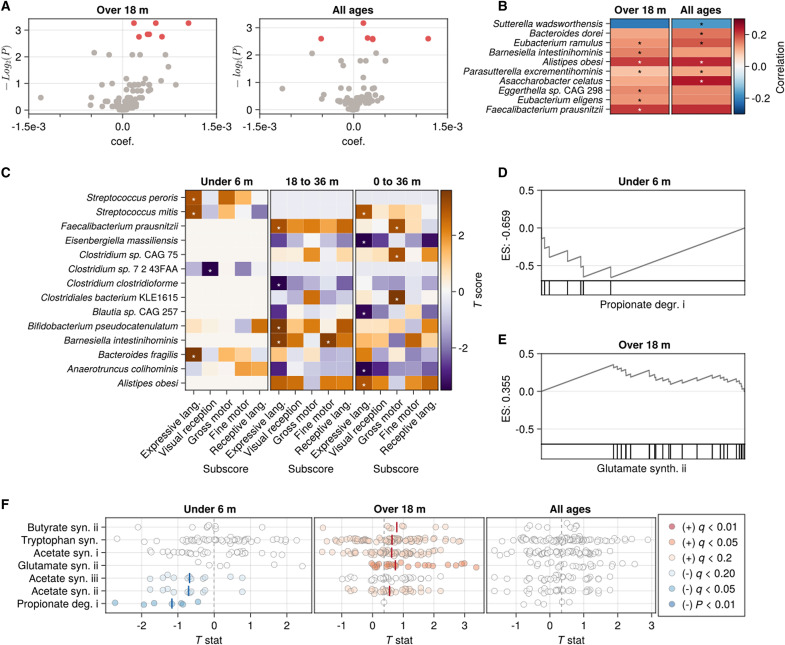
Taxa and gene functional groups are associated with cognitive function. (**A**) Volcano plot of multivariable linear model results showing the relationship between individual taxa and cognitive function score in children over 18 months of age (left) or across all ages (right), controlling for age, maternal education, and sequencing depth. Taxa that were significant after false discovery rate correction (*q* < 0.2) are colored red. (**B**) For taxa that were significantly associated with cognitive function, heatmaps of correlation with cognitive function in different age groups. (**C**) Multivariate linear models showing the relationship between individual taxa and domain-specific skill assessments from MSEL for children under 6 months (left), 18 to 36 months (right), or over across all ages (right) controlling for age, maternal education, and sequencing depth. Asterisks in (A) and (C) indicate significance (*q* < 0.2) after multiple hypothesis correction. (**D** and **E**) Enrichment plots for propionate degradation I gene set used in feature set enrichment analysis (FSEA) in children under 6 months (D) or over 18 months (E). (**F**) Summary of FSEA results in children under 6 months of age (left) over 18 months (middle) or across all ages (right), colored based on the significance of the association. Markers indicate the individual correlation of genes within a gene set, and vertical colored bars represent the median correlation of that gene set. Vertical dotted lines within each panel represent the median *T* statistic for all genes in that age group.

To assess whether individual microbial species were associated with cognitive function, we fit multivariable linear regression models (LMs) (*31*) for the relative abundance of each species that had at least 15% prevalence in a given age group (Fig. 2A; *N*_species_ = 116 for 0 to 120 months, *N*_species_ = 54 for 0 to 6 months, *N*_species_ = 136 for 18 to 120 months). No species were significantly associated (*q* < 0.20) with cognitive function in children under 6 months old after adjusting for age and maternal education. By contrast, in children over 18 months of age, several microbial species were significantly enriched in children with higher cognitive function scores (Fig. 2B), including *Alistipes obesi*, *Asaccharobacter celatus* [also known as *Adlercreutzia equolifaciens* subsp. *celatus* (*32*)], and SCFA-producing probiotic species such as *Eubacterium eligens* and *Faecalibacterium prausnitzii* (Fig. 2B) (*33*). *Sutterella wadsworthensis* was the only microbial species that was significantly negatively associated with cognitive function score (*q* = 0.165).

Cognitive assessments (MSEL, WPPSI, WISC) are composites of multiple subscales that assess different cognitive domains. While the composite scores are broadly analogous across ages and test modality, the subscales assessed in each test are different. To assess whether microbial taxa were associated with the development of specific cognitive domains, we fit multivariate linear models for each species present in at least 10% of samples for children under 6 months, 18 to 36 months, or all ages with a concurrent MSEL score (Fig. 2C). Several taxa associated with composite scores (Fig. 2B) were also associated with one or more subscores, including *F. prausnitzii* and *A. obesi*. While no taxa were significantly associated with composite scores in children under 6 months old, two species from the genus *Streptococcus* (*S. peroris* and *S. mitis*), as well as *B. fragilis*, were positively associated with expressive language in this age group. *S. mitis* was also associated with expressive language when looking at all ages, while *Eisenbergiella massiliensis*, *Anaerotruncus colihominis*, and an unclassified *Blautia* species were negatively associated.

Given that different microbial species might occupy the same metabolic niche in different individuals, we hypothesized that microbial genes grouped by functional activity would be associated with cognition. To test this, we performed feature set enrichment analysis (FSEA) on groups of genes with neuroactive potential (*9*) and concurrent cognitive ability score (Table 2 and Fig. 2, D to F) and found that several metabolic pathways were either significantly enriched or depleted in children with higher cognitive ability scores. For example, genes for degrading the 3-carbon SCFA, propionate, were significantly depleted in children with higher cognitive ability scores in children under 6 months [Table 2; propionate degradation I, enrichment score (ES) = −0.66, corrected *P* value (*q*) = 0.029], as were genes for synthesizing the 2-carbon SCFA acetate (acetate synthesis II, ES = −0.491, *q* = 0.059; acetate synthesis III, ES = −0.409, *q* = 0.133). In children over 18 m, these metabolic associations were reversed, with both acetate synthesis and propionate degradation being enriched in children with higher cognitive scores (acetate synthesis I, ES = 0.192, *q* = 0.145; acetate synthesis II, ES = 0.248, *q* = 0.15; propionate degradation I, ES = 0.154, *q* = 0.178), as was synthesis of the 4-carbon SCFA butyrate (butyrate synthesis II, ES = 0.414, *q* = 0.178). Synthesis of the amino acid glutamate was also significantly enriched in children with higher cognitive function scores in this age group (glutamate synthesis II, ES = 0.355, *q* = 0.023). Together, these results suggest that microbial metabolic activity, particularly the metabolism (synthesis and degradation) of neuroactive compounds, may have effects on cognitive development.

**Table 2.** Neuroactive microbial gene sets are associated with cognitive function Bold text indicates significant (*q* < 0.2) gene sets with a positive or negative pseudomedian.

Age group	0–6 months		18–120 months	
Gene set	ES	*q*	ES	*q*
Acetate syn. I	−0.165	0.284	0.192	**0.145**
Acetate syn. II	−0.491	**0.059**	0.248	**0.15**
Acetate syn. III	−0.409	**0.133**	0.215	0.209
Butyrate syn. II	−0.357	0.284	0.414	**0.178**
Glutamate syn. II	0.343	0.284	0.355	**0.023**
Propionate deg. I	−0.659	**0.029**	0.154	**0.178**

### Gut microbial taxonomic and functional profiles predict concurrently measured cognitive function

FSEA relies on understanding functional relationships between individual genes. However, because the relationships between individual taxa are still largely unknown, we turned to random forest (RF) models, a family of unsupervised nonparametric ML method that enables the identification of underlying patterns in large numbers of individual features (here, microbial species). Previous studies have reported successful use of RFs for processing highly dimensional and sparse data from the domain of genomics (*34*–*38*), along with other works where it was used to predict cognitive conditions related to Alzheimer’s disease in different scenarios (*39*, *40*). Given that gut microbial profiles, as well as neurocognitive development, may partially reflect socioeconomic and demographic factors, we assessed the performance of RF regressors where maternal education [here used as a proxy of socioeconomic status (SES)], sex, and age were included as possible predictors, either alone or in combination with microbial taxonomic profiles (Table 3).

**Table 3.** RF models on microbial taxa and genes can learn associations with concurrently measured cognitive performance. Benchmark metrics for the cognitive assessment score prediction models. Confidence intervals (CIs) are calculated from the distribution of metrics from repeated CV at a confidence level of 95. RMSE was used to estimate how well the random forest model was able to predict the outcome. Demo. column indicates whether demographic factors such as maternal education, sex, and age were included as predictors.

Subject ages (months)	Microbial feature	Demo.	Test set RMSE (±CI)	Test set correlation (±CI)
0–6	−	+	13.37 ± 0.05	−0.16 ± 0.01
	Taxa	−	12.99 ± 0.04	−0.11 ± 0.01
		+	13.01 ± 0.04	−0.13 ± 0.01
	Genes	−	12.95 ± 0.04	−0.05 ± 0.01
		+	12.95 ± 0.04	−0.05 ± 0.01
18–120	−	+	17.06 ± 0.04	0.511 ± 0.003
	Taxa	−	18.71 ± 0.04	0.347 ± 0.003
		+	18.25 ± 0.04	0.429 ± 0.003
	Genes	−	18.96 ± 0.04	0.299 ± 0.003
		+	18.74 ± 0.04	0.341 ± 0.003

**Fig. 3.**
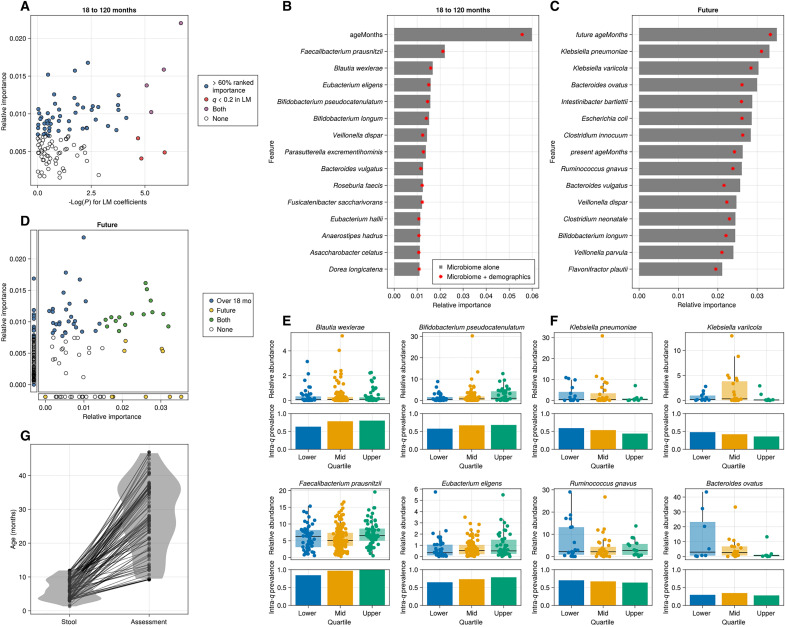
Random forest models predict both concurrent and future measured cognitive function. (**A**) Comparison of RF predictor importance versus linear models for children over 18 months. Colors represent whether the species belong to the group of top-important features that account for 60% of the cumulative importance on the RF model, if that species was significant (*q* < 0.2) in linear models, both, or neither. (**B**) Comparison of RF predictor importance for microbial features between concurrent and future cognitive function measure. Colors represent whether the species belong to the group of top-important features that account for 60% of the cumulative importance on the RF model only for concurrent prediction, only for the future prediction, or for both models. Features absent of one model are shown on the origin-aligned side plots. (**C**) Distribution of ages for each microbiome sampling—cognitive assessment dyad. Blue violin plot shows distribution of sampling age, while red violin plot shows distribution of assessment age. Black lines connect ages for each individual subject. Ranked feature importance for taxa in RF models for both concurrent (**D**) and future (**E**) cognitive function measurement prediction. Red dots indicate how relative importances change (among taxa) when demographics (sex, SES) are used as covariables. Abundances and prevalences of taxa that are important for RF models for both concurrent (**F**) and future (**G**) assessment prediction.

As with LMs, RF models for children under 6 months old performed poorly [mean test set correlation −0.13, mean root mean square error (RMSE) 13.01], but RFs were consistently able to learn the relationship between taxa and cognitive function scores in children over 18 months of age (mean test set correlation 0.429, mean RMSE 18.25). Species that were important in RF overlapped with those that were significant when testing the relationship with LMs (Fig. 3A and table S1). Both analyses identified *F. prausnitzii*, *E. eligens*, *Parasutterella excrementihominis*, and *A. obesi* as important (*q* < 0.2 for LMs, cumulative importance <60% for RFs). Other taxa that were significant in LMs were not identified by RF models including *Eggerthella* sp. CAG 298 and *Barnesiella intestinihominis*. *Eubacterium ramulus* was just outside our cutoff (64% cumulative importance). RF models also identified an additional 48 species that were important for discriminating cognitive function scores (Table 3 and Fig. 3, B and E). Subject age was consistently ranked highly in feature importance, which could indicate that decision branches based on microbial taxa have increased purity when considering the subject’s age or that age itself is a useful predictor.

### Gut microbial profiles in the first year of life predict future cognitive function

While LMs did not identify any taxa in the 0- to 6-month range that were associated with concurrently measured cognitive function, we reasoned that developmental trajectories that begin in utero may not be immediately altered by microbial exposures such that microbial influences early in life may take time to manifest. To test this hypothesis, we asked whether RF models trained on taxonomic profiles in the first year of life could predict future cognitive performance measured at least 6 months after the stool sample was collected (Fig. 3, C, D, and F, and Table 4). Models were consistently able to learn microbial species that are associated with cognitive performance at a future visit (mean time between visits: 21.5 months, SD = 9.9 months; Fig. 3G, mean test set correlation: 0.27).

**Table 4.** RF models on microbial taxa and genes can learn associations with future cognitive performance. CIs are calculated from the distribution of metrics from repeated CV at a confidence level of 95. RMSE was used to estimate how well the random forest model was able to predict the outcome. Demo. column indicates whether demographic factors such as maternal education, sex, and age were included as predictors.

Microbial feature	Demo.	Test set RMSE (±CI)	Test set correlation (±CI)
−	+	20.25 ± 0.07	0.43 ± 0.0
Taxa	−	22.32 ± 0.08	0.07 ± 0.0
	+	21.53 ± 0.08	0.27 ± 0.0
Genes	−	22.21 ± 0.08	0.12 ± 0.0
	+	22.08 ± 0.08	0.15 ± 0.0

Nearly all important taxa in RF models that predicted cognitive function in children older than 18 months were also important in models that predicted future cognitive function, if they were present (Fig. 3, B and C, and table S5). At the same time, many additional species were important for future prediction models that were not important in the models measuring concurrent cognitive function. In particular, feature importance for future prediction models was dominated by two species of *Klebsiella*, *K. pneumoniae* and *K. varicola* (Fig. 3, C and F). Many of the other species that ranked highest in feature importance were also opportunistic pathogens, or previously reported to be linked with disease (e.g., *Ruminococcus gnavus*, *Escherichia coli*).

### Gut microbial taxa are associated with specific cognitive functions

**Fig. 4.**
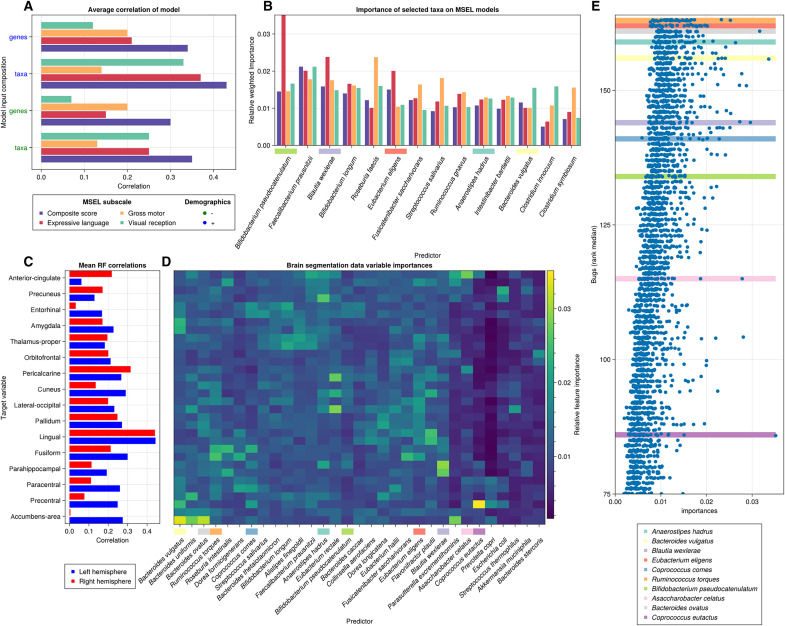
Coupled importance of microbes in neuroanatomy and subscores are consistent with known subcortical associations with cognition. (**A**) Test set correlations for RF models predicting MSEL subscale scores, with or without demographics included as predictors. (**B**) Important taxa in models predicting MSEL subscale scores. (**C**) Average test set correlations for prediction of MRI segmentation data from microbiome and demographics on select segments after repeated cross-validation. (**D**) Heatmap of average individual relative taxa importances on each brain segment. Importances are reported as proportions relative to the sum of importances for each model, since every model is trained with 132 features, and even distribution of importance would be 0.75% for each feature. (**E**) Segments and taxa ordered by hierarchical clustering analysis on a select list of species with high ranks on average importances, and their respective highest-load segments.

Given that the development of different domains of cognitive function (e.g., gross motor development versus visual perception) may develop at different times and be affected by different molecular signals, we sought to determine whether the associations of microbial taxa with cognitive function are general across all cognitive subdomains. To accomplish this, we assessed individual subscales of MSEL (birth to 36 months; see Materials and Methods). Taxonomic profiles coupled with demographics exhibited predictive potential toward three of five probed subscales (Fig. 4A and Table 5), namely, expressive language (test set RMSE 12.05, test set correlation 0.368), gross motor (test set RMSE 11.07, test set correlation 0.137), and visual reception (test set RMSE 15.2, test set correlation 0.333). Between the subscales, both the predictability (measured by the test set figures of merit) and the relative importances of several taxa (Fig. 4B) is variable. Although certain species such as *B. longum* and *F. prausnitzii* have similar relative importance across all subscales, others are substantially different (tables S2 to S4). Notably, *Bifidobacterium pseudocatenulatum*, *Blautia wexlerae*, and *E. eligens* are substantially more important for the prediction of expressive language, while *Roseburia faecis*, *Streptococcus salivarius*, and *Fusicatenibacter saccharivorans* are distinctly predictive of gross motor, and *Clostridium innocuum* and *Bacteroides vulgatus* stand out on models targeting visual reception (tables S2 to S4).

**Table 5.** RF models on microbial taxa and genes can learn associations with performance in cognitive subdomains. Benchmark metrics for the Mullen subscales prediction models. CIs are calculated from the distribution of metrics from repeated CV at a confidence level of 95. Root mean square error (RMSE) was used to estimate how well the random forest model was able to predict the outcome. Demo. column indicates whether demographic factors such as maternal education, sex, and age were included as predictors. Age range, in all cases, was 18 to 120 months.

Target Mullen scale	Microbial feature	Demo.	Test set RMSE (±CI)	Test set correlation (±CI)
Expressive Language	−	+	11.17 ± 0.04	0.48 ± 0.0
	Taxa	−	12.37 ± 0.04	0.25 ± 0.0
		+	12.05 ± 0.04	0.37 ± 0.0
	Genes	−	12.59 ± 0.04	0.15 ± 0.0
		+	12.46 ± 0.04	0.21 ± 0.0
Gross motor	−	+	10.53 ± 0.04	0.346 ± 0.006
	Taxa	−	11.08 ± 0.04	0.134 ± 0.006
		+	11.07 ± 0.04	0.137 ± 0.006
	Genes	−	10.98 ± 0.04	0.197 ± 0.005
		+	10.98 ± 0.04	0.197 ± 0.005
Visual reception	−	+	14.13 ± 0.04	0.462 ± 0.003
	Taxa	−	15.49 ± 0.04	0.245 ± 0.004
		+	15.2 ± 0.04	0.333 ± 0.004
	Genes	−	15.97 ± 0.04	0.067 ± 0.004
		+	15.84 ± 0.04	0.118 ± 0.005

### Gut microbial taxonomic profiles predict brain structure differences

Given that individual microbes could be associated with particular domains of cognitive function and not others, if there are causal effects of microbial metabolism on cognitive function, they might be reflected in changes in neuroanatomy. We again used RF modeling to associate gut taxonomic profiles with individual brain regions identified in MRI scans (children over 18 months old, *N* = 124). Some brain regions were more readily predicted by RF models trained on microbial taxa (Tables 5 and 6 and table S6), in particular those that were highly correlated with age. These included the L/R lingual gyrus (mean RF correlations, left = 0.444, right = 0.441; importance for age, left = 6.8%, right = 7.7%) and the L/R pericalcarine cortex (mean RF correlations, left = 0.267, right = 0.315; importance for age, left = 3.6%, right = 6.2%). In many cases, however, age was not an important variable in high-performing models, such as that for the left accumbens area (mean RF correlations = 0.275; relative age importances = 1.1%).

**Table 6.** RF models on microbial taxa predicting brain regions have generally good performance. Mean absolute proportional error (MAPE) and correlation coefficient (*R*) of test set predictions are reported across models for all brain regions. Complete list of values can be found in table S6.

Statistic	MAPE	*R*
Mean	0.073	0.146
Standard deviation	0.029	0.131
Maximum	0.205	0.598
75th percentile	0.081	0.212
50th percentile	0.066	0.129
25th percentile	0.057	0.073
Minimum	0.036	−0.133

While models for many regions were highly concordant across the left and right hemispheres in terms of both model performance and microbial feature importance, other regions had substantial bilateral differences. For example, the left accumbens area has one of the highest test set correlations of our brain region models (*R* = 0.288), as compared to the right, which had a negligible test set correlation (*R* = 0.005). Feature importance for models of the left accumbens area was dominated by three species of *Bacteroides*, *B. vulgatus* (3.3% importance), *B. ovatus* (3.2% importance), and *B. uniformis* (2.8% importance).

While RF models for multiple brain regions had many important microbial taxa, others were dominated by a small number of taxa, many of which were also identified in LMs and RF models of cognitive function (Figs. 2 and 3). In general, for all segments, a consistent number of species between 28 and 42 (median = 38, less than 30% of the input features) were responsible for half of the cumulative importance, regardless of model performance (Fig. 4, C to E, and table S2). We observed two major patterns of importance distribution from species over the brain segments; some species portrayed high contributions to multiple different segments, while others contributed modestly to just one or two brain segments. Notable cases of the first pattern included nine species: *Anaerostipes hadrus*, *B. vulgatus*, *B. ovatus*, *F. saccharivorans*, *Ruminococcus torques*, *Eubacterium rectale*, *Coprococcus comes*, *A. celatus*, and *B. wexlerae*, which, combined, account for approximately 10% of the cumulative relative importances, computed after fitness weighting on the reported brain segments (Fig. 4, D and E).

Among these, *A. hadrus* is the variable with highest average relative importance along all reported models (mean = 1.4%; min = 0.8%; max = 3.6%; Fig. 4, D and E). *B. wexlerae*, which was important in RF models of cognitive function, was also important in models of the entorhinal, fusiform, and lingual areas and had the highest importance in models predicting the size of both the left and right parahippocampus. Another cluster of model importance contained taxa associated with the basal forebrain, especially the cingulate and the accumbens. This cluster contains *B. ovatus* and *B. uniformis*, but also *Alistipes finegoldii and S. salivarius*.

*A. celatus*, an equol-producing species, is an example of the second contribution pattern, where a taxon only contributes significantly to a very small number of segments. This species was highly important in predicting the relative volume of the right anterior cingulate (2.8% relative importance). Another example of this pattern is *Coprococcus eutactus*, which is heavily associated with the left precentral area (3.5% importance). Finally, *C. comes* has an importance distribution that splits almost evenly among the two previously reported clusters. Its loading on the prediction of the left posterior cingulate is the highest for a microbe in RF models (3.7% importance); only age had higher relative importance in any model. At the same time, *C. comes* is one of the most important predictors for neighboring areas of the posterior cingulate such as the pars opercularis (importances, left = 1.9%, right = 2.5%), along with upper regions like the left precentral (2.0% importance) and paracentral lobes (importances, left = 2.0%, right = 1.5%).

## DISCUSSION

The relationship between the gut microbiome and brain function via the gut-microbiome-brain axis has gained increasing acceptance largely as a result of human epidemiological studies investigating atypical neurocognition [e.g., anxiety and depression, neurodegeneration, attention deficit/hyperactivity disorder, and autism (*41*, *42*)] and mechanistic studies in animal models (*27*, *43*). The results from these studies point to the possibility that gut microbes and their metabolism may be causally implicated in cognitive development, but this study is the first to our knowledge that directly investigates microbial species and their genes in relation to typical development in young, healthy children. Understanding the gut-brain-microbiome axis in early life is particularly important, since differences or interventions in early life can have outsized and longer-term consequences than those at later ages due to the dynamic and plastic nature of both the gut microbiome and the brain. Further, even in the absence of causal impacts of microbial metabolism, identifying risk factors that could point to other early interventions would also have value.

We identified several species in the family Eggerthelaceae that were associated with cognitive function, including *A. celatus* [a subspecies of *A. equolofaciens* (*44*)] and *Eggerthella lenta*. Many members of this family are known in part due to unique metabolic activities. For example, *A. equolofaciens* produces the nonsteroidal estrogen equol from isoflaven found in soybeans (*45*). This is particularly intriguing, since *A. celatus* was also the most important features in RF models of the right anterior cingulate, which has been linked to social cognition and reward-based decision making (*46*–*48*), and estrogen has previously been shown to have activity in this brain region (*49*). Another Eggerthelaceae, *Gordonibacter pamelaeae*, also has a unique metabolism that may affect the brain, as it can metabolize the polyphenol ellagic acid (found in pomegranates and some berries) into urolithin, which has been shown in some studies to have a neuroprotective effect (*50*, *51*). Although *G. pamelaeae* was not identified as an important feature in RF models, it was also relatively low prevalence in our dataset, occurring in only 5% of samples at greater than 2% abundance (in contrast to *A. celatus*, which was in 32% of samples). While we could not find published evidence of neurological roles for *E. lenta*, it has been extensively studied for its ability to metabolize drug compounds such as the plant-derived heart medication digoxin (*52*). The metabolic versatility of this clade and the large number of species that are associated with cognition make these microbes prime targets for further mechanistic studies.

We did not identify any statistical associations between individual species and cognitive performance in our youngest subset (0 to 6 months). We hypothesize that this may be because brain developmental trajectories begin in utero, and microbial influences (that can only begin at birth) take additional time to manifest. In line with this hypothesis, RF models were able to learn associations between early-life microbiomes and future cognitive function scores (Fig. 3). The top-ranked species for these models included several taxa that were important for predicting concurrent cognitive assessment scores after 18 months, notably pathobionts in the family *Enterobacteriaceae* such as *Escherichia* and *Klebsiella*. Representatives of these genera are known to be early and long-term persistent colonizers of infant intestine that can, in some settings, promote inflammatory response (*53*). By contrast, several inflammation-reducing representatives of the *Bifidobacterium* genus, from the early human milk oligosaccharide–metabolizer *B. longum* to the later complex carbohydrate relying *B. pseudocatenulatum* (*54*), commonly associated with weaning to solid food in early age, also showed high relative importance. The involvement of most important taxa in different aspects of inflammatory response resonates with previous findings that associate chronic inflammation with changes to neural responses in some types of stimuli (*55*).

Other bacteria that were associated with both cognitive ability and brain structure had also have possible connections to previous studies. For example, RF models predicting the size of the left nucleus accumbens were dominated by *Bacteroides* species, especially *B. vulgatus*, *B. uniformis*, and *B. ovatus*. *B. ovatus* has been shown to produce a number of neuroactive compounds including SCFAs and neurotransmitters such as γ-aminobutyric acid (GABA) (*56*). Another *Bacteroides* species, *B. fragilis*, was able to ameliorate autism spectrum disorder (ASD)–like symptoms in a mouse model by consuming a microbially dependent metabolite 4-ethylphenylsulfate (4EPS) (*43*), while *B. ovatus* is a primary producer of a precursor to 4EPS (*27*) and increased anxiety behaviors in mice. The fact that the importance of these species in RF models and the high performance of RF models in predicting this region were unilateral (affecting the left but not the right accumbens area) was also notable. Most healthy individuals have a rightward asymmetry in the nucleus accumbens, which is involved in reward learning and risk taking behaviors (*57*, *58*). Reduced asymmetry between right and left nucleus accumbens has been associated with substance use disorder in young adults (*59*). The accumbens area is also associated with reward control, and in individuals diagnosed with attention deficit hyperactivity disorder (ADHD), it has been shown to have a divergent neuromorphology (*60*).

Our ability to probe individual cognitive subscales provided results that also align with previous findings and provide insight to potential gut-microbial connections between cognition and neuroanatomy. For example, a longitudinal neurocognitive development study pointed at bilateral associations between the thalamus and the expressive language component of the Mullen scales (*61*). In line with this finding, we found that taxa such as *B. wexlerae* and *A. hadrus* were among the top 15% important predictors on both left and right thalamus and the expressive language subscale. Another example of those findings is a previously reported association between the central opercular cortex (COC) and gross motor development (*62*). In this case, we identified that butyrate-producing *F. saccharivorans* is among the 15% most important predictors for both the gross motor subscale and three bilateral components of the COC (left and right pars orbitalis, pars opercularis and pars triangularis). Previous results also link to relationships between the caudate and the cerebellum, and the visual reception score (*63*). *S. salivarius* was consistently found among the top 20% predictors for this subscale, along with both sides of the thalamus and cerebellum white matter, along with cerebellar vermal lobules I to X.

As is made apparent by the potentially opposing roles of *B. fragilis* and *B. ovatus*, individual species from the same genus may play different roles. As such, the use of shotgun metagenomic sequencing may be crucial to identifying microbial influences on the brain, since it enables species-level resolution of microbial taxa. A previous study of cognition in 3-year-old subjects used 16*S* rRNA gene amplicon sequencing and showed that genera from the Lachnospiraceae family as well as unclassified Clostridiales (now Eubacteriales) were associated with higher scores on the Ages and Stages Questionnaire (*64*). However, each of these clades encompass dozens of genera with diverse functions, each of which may have different effects. Several of the taxa that were positively associated with cognitive function in this study, including *B. wexlerae*, *Dorea longicatena*, *R. faecis*, and *A. finegoldii*, are Clostridiales, as is *R. gnavus*, which we found was negatively associated with cognitive function (Fig. 3, C and F). This kind of species-level resolution is typically not possible with amplicon sequencing.

In addition to improved species-level resolution, shotgun metagenomic sequencing also enables gene-functional insight. We showed that genes for the metabolism of SCFAs, both their degradation and synthesis, are associated with cognitive function scores (Fig. 2). SCFAs are produced by anaerobic fermentation of dietary fiber and have been linked with immune system regulation as well as directly with brain function (*65*). Microbial metabolism of other potentially neuroactive molecules was also associated with cognitive ability. For example, we were particularly interested in genes for metabolizing glutamate, which is a critical neurotransmitter controlling neuronal excitatory/inhibitory signaling along with GABA, and their balance in the brain controls neural plasticity and learning, particularly in the developing brain (*66*, *67*). However, while the differential abundance of genes for the metabolism of neuroactive compounds like these is suggestive, it is difficult to reason about the relationship between levels of these genes and the gut concentrations of the molecules their product enzymes act on since the presence or absence of metabolites may select for microbes with the ability to degrade or produce that metabolite, respectively. For this reason, future studies coupling shotgun metagenomics with stool metabolomics could improve our understanding of the relationship between microbial metabolism and cognitive development. Serum metabolomic analysis may be even more informative, since the metabolites measured in stool are not necessarily indicative of exposure, but drawing sufficient blood from children, especially very young children, is challenging. Further, strain-level analysis linking specific gene content in species of interest could further refine targeted efforts at identifying specific metabolic signatures of microbe-brain interactions.

The use of multiple age-appropriate cognitive assessments that could be normalized to a common scale enabled us to analyze microbial associations across multiple developmental periods, but carries several drawbacks. In particular, the test-retest reliability, as well as systematic differences between test administrators, may introduce substantial noise into these observations, particularly in the youngest children. In addition, our study period overlapped with the beginning of the coronavirus disease 2019 (COVID-19) pandemic, and we and others have observed some reduction in measured scores for children that were assessed after the implementation of lockdowns. In our subject set for this study, these effects are more pronounced in some age groups due to the period when active recruitment for the study was occurring (fig. S3) (*68*, *69*).

This analysis allowed us to establish links between microbial taxa and their functional potential with cognition and brain structure. Although we did not directly test the causal relationships between gut microbial taxa and their genes, the gut, and the brain, this study provides clear and statistically significant associations that could serve as targets for future efforts in preclinical models. For example, mouse models of brain development, learning, and executive function could be assessed in germ-free animals with the addition of metabolically characterized and manipulated microbes. Future studies should also focus on characterizing the early-life microbiome and neurocognitive development across different geographic regions and lifestyles such as covering traditionally understudied populations, like low-resource urban, peri-urban, and rural communities, to obtain a more comprehensive understanding of the variability within the different gut microbiomes reflected on neurocognition. These studies would also provide us with the wealth of data on different strains from the same species to better understand the effect of genes and their products. Furthermore, culturing and microbial community enrichment studies combined with genetic manipulation and genomic approaches to understand microbial metabolism at the molecular level is key, as the metabolic functions shape and influence the human host and its health. The discovery of the neuroactive metabolites could provide us with biomarkers for early detection or necessary medicinally useful molecules that can be applied in intervention.

## MATERIALS AND METHODS

### Study ethics

All procedures for this study were approved by the local institutional review board at Rhode Island Hospital (Investigating Early Childhood Neurodevelopment 1500991, and Prenatal Influences on Fetal and Infant Brain Development 1501119; most recent approval for both: 15 January 2020), and all experiments adhered to the regulation of the review board. Written informed consent was obtained from all parents or legal guardians of enrolled participants.

### Participants

Data used in this study were drawn from an ongoing longitudinal brain and cognitive development, termed RESONANCE, based in Providence, RI, USA, and spanning fetal through adolescent development. The RESONANCE cohort is part of the National Institutes of Health (NIH) Environmental influences on Child Health Outcomes (ECHO) study (*70*, *71*) and includes neuroimaging (MRI), neurocognitive assessments, biospecimen analyses, subject genetics, broad environmental exposure data (such as chemical, water quality, and nutrition), and rich demographic, socioeconomic, family, and medical history information.

As a study of neurotypical development, mothers and their children exclusion criteria included major risk factors for abnormal or atypical development, i.e., preterm birth (<37 weeks gestation), low birth weight (<1500 g), in utero exposure to alcohol, cigarette or illicit substance exposure; abnormalities on fetal ultrasound; non-singleton or complicated pregnancy, including preeclampsia, high blood pressure, or gestational diabetes; complicated vaginal or cesarian birth; 5 min Apgar scores <8; NICU admission; neurological disorder in the child (e.g., head injury resulting in loss of consciousness, epilepsy); and psychiatric or learning disorder (including maternal depression) in the infant, parents, or siblings requiring medication in the year before pregnancy. In addition to screening at the time of enrollment, on-going screening for worrisome behaviors using validated tools was performed to identify at-risk children and remove them from subsequent analysis. For this study, 381 typically developing children between the ages of 40 days and 10 years (median age 2 years, 2 months) were selected for analysis based on having provided at least one stool sample in the same week as being evaluated using one of the cognitive ability assessments (MSEL, WPPSI, WISC-V).

### Cognitive assessments

General cognitive ability was assessed using age-appropriate performance-based measures. For children under 3 years of age, we used the Early Learning Composite (ELC) from MSEL (*22*). MSEL is a standardized and population-normed tool that assesses function in five major domains: fine and gross motor, expressive and receptive language, and visual functioning. The ELC is an age-normalized composite derived from these individual domains with a mean of 100 and SD of 15. For older children, full-scale IQ (FSIQ) was assessed using WPPSI-4 (children 3 to 6) (*23*), and WISC-V for children older than 6 (*24*). Like the ELC, FSIQ is normalized to a mean of 100 and SD of 15.

### Stool sample collection and sequencing

Stool samples (*n* = 493) were collected by parents in OMR-200 tubes (OMNIgene GUT, DNA Genotek, Ottawa, Ontario, Canada), stored on ice, and brought within 24 hours to the laboratory in RI where they were immediately frozen at −80°C. Stool samples were not collected if the subject had taken antibiotics within the last 2 weeks. DNA extraction was performed at Wellesley College (Wellesley, MA). Nucleic acids were extracted from stool samples using the RNeasy PowerMicrobiome kit, excluding the DNA degradation steps. Briefly, the samples were lysed by bead beating using the Powerlyzer 24 Homogenizer (Qiagen, Germantown, MD) at 2500 rpm for 45 s and then transferred to the QIAcube (Qiagen, Germantown, MD) to complete the extraction protocol. Extracted DNA was sequenced at the Integrated Microbiome Resource (IMR; Dalhousie University, NS, Canada).

Shotgun metagenomic sequencing was performed on all samples. A pooled library (maximum 96 samples per run) was prepared using the Illumina Nextera Flex Kit for MiSeq and NextSeq from 1 ng of each sample. Samples were then pooled onto a plate and sequenced on the Illumina NextSeq 550 platform using 150 + 150 base pair paired-end “high output” chemistry, generating 400 million raw reads and 120 Gb of sequence per plate.

### Computational analysis of metagenomes

Shotgun metagenomic sequences were analyzed using the bioBakery suite of computational tools (*72*). First, KneadData (v0.10.0) was used to perform quality control of raw sequence reads, such as read trimming and removal of reads matching a human genome reference. Next, MetaPhlAn (v3.1.0, using database mpa_v31_CHOCOPhlAn_201901) was used to generate taxonomic profiles by aligning reads to a reference database of marker genes. Finally, HUMAnN (v3.6.0) was used to functionally profile the metagenomes.

### ML for cognitive development

Prediction of cognitive scores was carried out as a set of regression experiments targeting real-valued continuous assessment scores. Different experiment sets were designed to probe how different representations of the gut microbiome (taxonomic profiles, functional profiles encoded as ECs) would behave, with and without the addition of demographics (sex and maternal education as a proxy of SES) on participants from different age groups.

For the concurrent cognitive assessment score predictions, all viable datapoints containing age at data collection, demographics, stool metagenomics, and cognitive assessment were used. The cognitive assessments were collected as either composite scores (for the experiments in Fig. 3) or, when available, subscores on MSEL (Fig. 4, A and B: expressive language, gross motor and visual reception). For the concurrent prediction experiments, when multiple viable longitudinal samples were present, the single earliest viable datapoint inside the age range of the experiment was considered. For the future prediction experiments, every demographics-complete subject contributed a single datapoint built from the combination of the latest metagenome collected within the stool collection time range and the latest future cognitive assessment on the assessment time range. Age (in months) was provided as a covariate for all models (table S3).

RFs (*73*) were selected as the prediction engine and processed using the DecisionTree.jl (*74*) implementation, inside the MLJ.jl (*75*). Independent RFs were trained for each experiment, using a set of default regression hyperparameters from Breiman (*73*), on a repeated cross-validation approach with different random number generator (RNG) seeds. One hundred repetitions of threefold cross-validation (CV) with 10 different intra-fold RNG states each were used, for a total of 3000 experiments per input set.

After the training procedures, the RMSE for cognitive assessment scores and mean absolute proportional error (MAPE) for the brain segmentation data, along with Pearson’s correlation coefficient (*R*), were benchmarked on the validation and train sets. MAPE was chosen as the metric for brain segments due to magnitude differences between median volumes of each segment, which would hinder interpretation of raw error values without additional reference.

To derive biological insight from the models, the covariate variable importances for all the input features, measured by mean decrease in impurity (or Gini importance), were also analyzed. Leveraging the distribution of results from the extensive repeated cross-validation experiments, rather than electing a representative model or picking the highest validation set correlation, a measure of model fitness (Eq. 1) was designed to weight the importances from each trained forest, and all “importance” values reported refer to the average fitness-weighted importance across all models. The objective was to give more weight to those with higher benchmarks on the validation sets (or higher generalizability) while penalizing information from highly overfit models, drawing inspiration from the approach used on another work using repeated CV on RFs with high-dimensional, low–sample size datasets (*76*). The resulting fitness-weighted importances were used to generate the values in Figs. 3 and 4.

$$\mathrm{fitness}=\sqrt{\max(r_{\mathrm{train}},0)\cdot \max(r_{\mathrm{test}},0)}$$ (1)

### MRI/segmentation

T1-weighted anatomical MRI data were acquired using a three-dimensional MP-RAGE sequence on 3T Siemens Trio scanner with the following parameters: echo time = 5.6 ms, repetition time = 1400 ms, inversion time = 950 ms, flip angle = 15 degrees, and 1.1 × 1.1 × 1.1 mm resolution. Image matrix and field of view were adjusted by infant size to achieve the desired image resolution. For children under 4 years of age, MRI was performed during natural nonsedated sleep using previously described methods (*77*). Older children were scanned awake while watching a movie or favorite TV show. To help minimize motion, pediatric (MedVac) immobilizers were used along with foam cushions and padding. Image quality was monitored during and following scanning. If motion artifacts were observed (e.g., ghosting, blurring, or other signal variations), scans were repeated or removed from analysis.

Following data acquisition, brain region volumes corresponding to total white and gray matter, cortical gray matter, brainstem, and left and right hemisphere lateral ventricles, thalamus, caudate, putamen, pallidum, hippocampus, amygdala, and nucleus accumbens were calculated using an atlas matching approach (*78*). Individual images were first nonlinearly aligned to Montreal Neurological Institute (MNI)–space using a multistep registration approach [ANTs 2.2 toolbox (*79*)]. The MNI-aligned Harvard-Oxford brain atlas was then aligned and superimposed onto the individual child by applying the inverse transformation, and the brain regions of interest were delineated (segmented) and their volume was calculated by summing the number of image voxels within each (*80*). Brain region volumes used in RF models were normalized to total brain volume (the sum of white and gray matter).

## Supplementary Materials

### This PDF file includes:

The Resonance Consortium Figs. S1 to S7 Tables S1 to S7
