# Supplementary material for: Gut-resident microorganisms and their genes are associated with cognition and neuroanatomy in children
Source: Sci Adv. 2023 Dec 22;9(51):eadi0497. doi: 10.1126/sciadv.adi0497 (PMC10745691; doi:10.1126/sciadv.adi0497)
Supplement: Supplementary file 1 — The Resonance Consortium Figs. S1 to S7 Tables S1 to S7 [file sciadv.adi0497_sm.pdf]

Science Advances
AAAS

Supplementary Materials for

Gut-resident microorganisms and their genes are associated with cognition and neuroanatomy in children

Kevin S. Bonham *et al.*

Corresponding author: Vanja Klepac-Ceraj, vklepacc@wellesley.edu

Sci. Adv. **9**, eadi0497 (2023)
DOI: 10.1126/sciadv.adi0497

This PDF file includes:

The Resonance Consortium
Figs. S1 to S7
Tables S1 to S7

The Resonance Consortium

The RESONANCE Consortium Consists of the following individuals:

Authors: Sean CL Deoni[1], Viren D'Sa[1], Muriel Bruchhage[1], Jennifer Beauchemin[1], Rosa Cano[1], Vanja Klepac-Ceraj[2], Kevin Bonham[2], Shelley McCann[2]

Collaborators: Susan Carnell[4], Elena Jansen[4], Jennifer R Sadler[4], Gita Thapaliya[4], Matthew Huentelman[3], Candace Lewis[3], Matthew D. De Both[3], Marcus A. Naymik[3], Jessica Fernandes[1], Elizabeth Walsh[1], Brittany Rhodes[1], Caroline Wallace[1], John Rogers[1], Alexandra Volpe[1], Monique LeBourgeois[5], Hans Georg Mueller[6], Jane-Ling Wang[6], Changbo Zhu[6] Yaqing Chen[6], Joseph Braun[7]

1. Department of Pediatrics, Warren Alpert Medical School at Brown University, Providence RI, USA
2. Department of Biological Sciences, 106 Central Street, Wellesley College, Wellesley MA 02445, USA
3. The Translational Genomics Research Institute, Neurogenomics Division, 445 N Fifth Street, Phoenix, AZ 85004 USA
4. Department of Psychiatry and Behavioral Sciences, Johns Hopkins University School of Medicine, Baltimore, MD, USA
5. Department of Integrative Physiology, University of Colorado, Boulder. CO, USA
6. Department of Statistics, University of California, Davis
7. School of Public Health, Brown University, RI, USA

Supplementary Figures and Tables

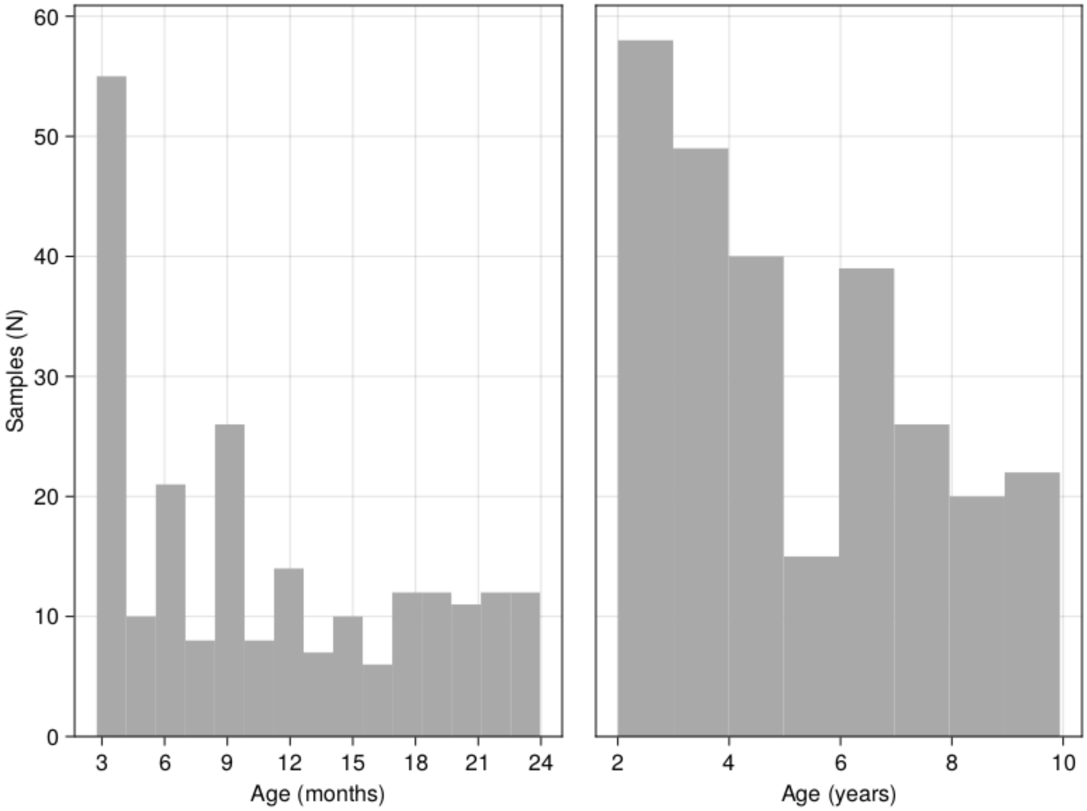

Figure S1: The age of participants when samples were collected is weighted towards early times. Sample collection by age - related to Figure 1A. A histogram showing the number of samples included in this study by the age of the child when the sample was collected.

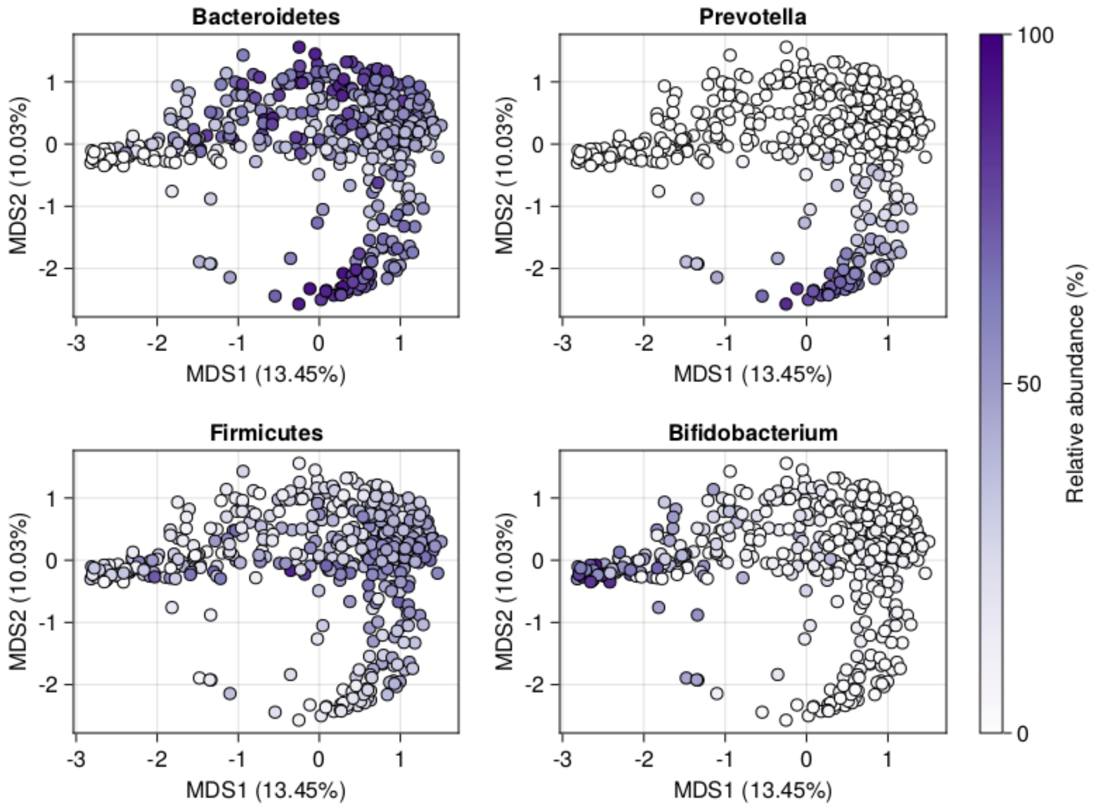

Figure S2: The broad pattern of microbial beta diversity is consonant with previous studies. Principal coordinates analysis of taxonomic profiles - related to Figure 1C. PCoAs are colored by the relative abundance per-sample of major phyla (Bacteroidetes, top left; Firmicutes, bottom left) and genera (*Prevotella*, top right; *Bifidobacterium*, bottom right).

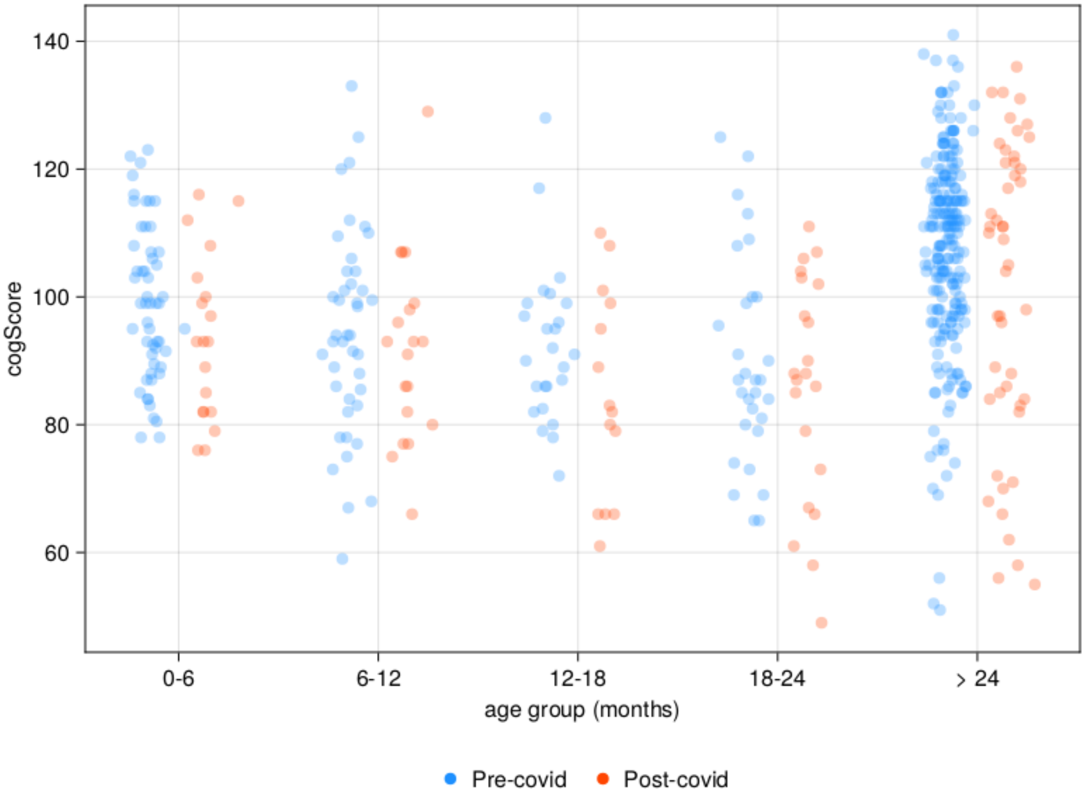

Figure S3: The mean and variance of cognitive assessment scores differs between participant age brackets, partially in association with COVID-19. Scores in red were collected after March of 2020.

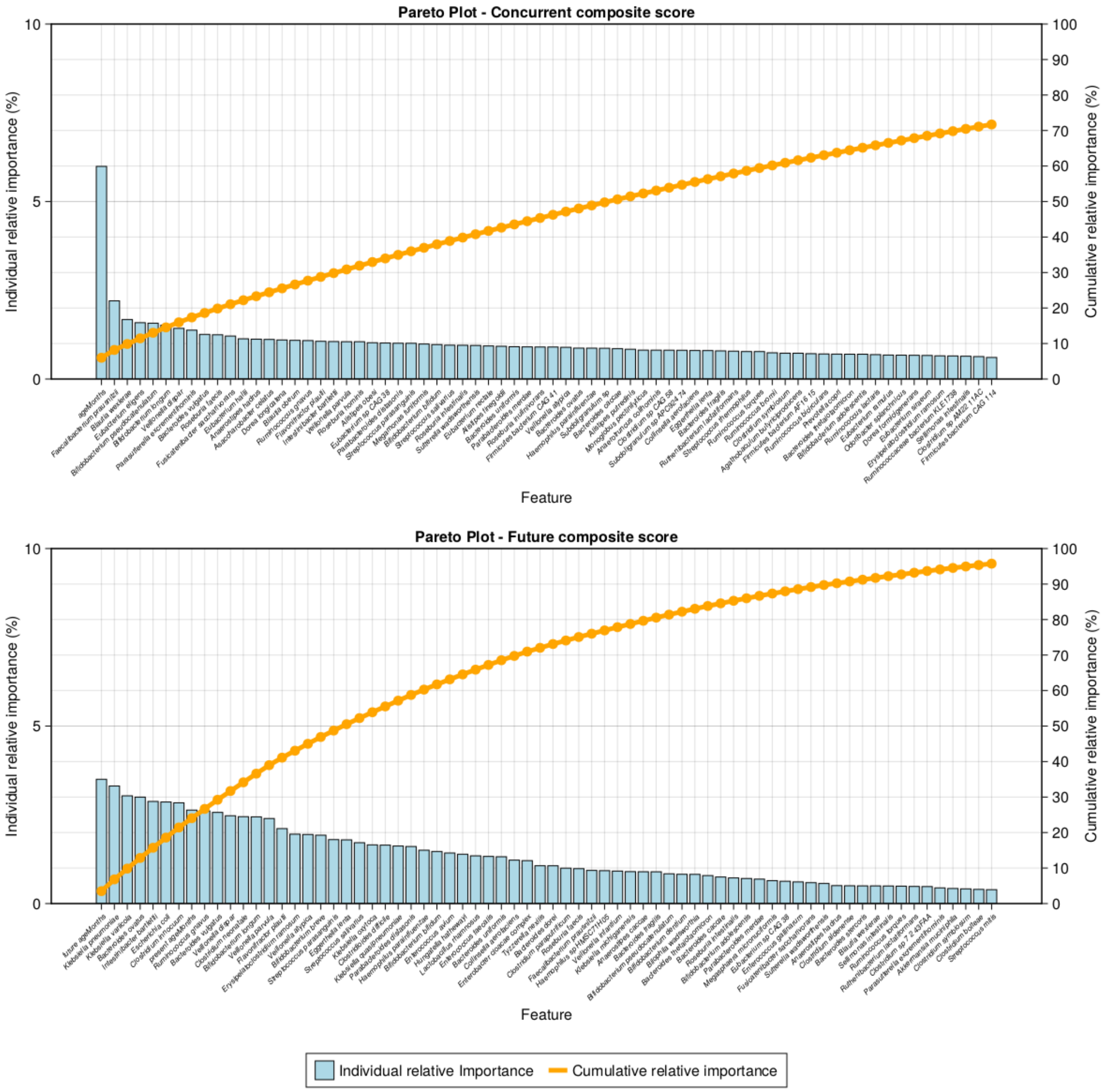

Figure S4: A small subset of species explains a large amount of the cumulative relative importance on both concurrent and future cognitive prediction models. Pareto plots for RFs of cognitive function - related to Figure 3C-D. The cumulative importance (orange line), as well as ranked importance (blue bars) of features (species) in models of cogntive performance score in children from birth to 6 months (top) or in children from 18 to 120 months (bottom).

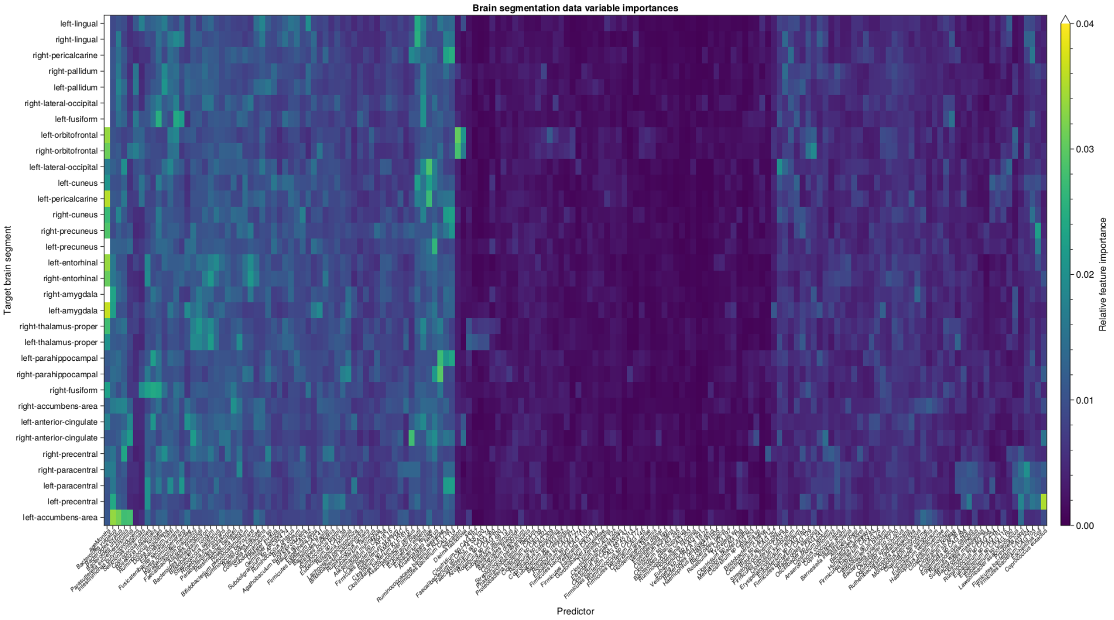

Figure S5: Cluster analysis on importances of the full microbial set for neuroanatomy prediction reveals different patterns of loadings on prediction models. Heatmap of feature importances for each brain region - related to Figure 4B. This heatmap contains all features included in models (including age) and all brain segments. Importance values over 0.04 are colored white.

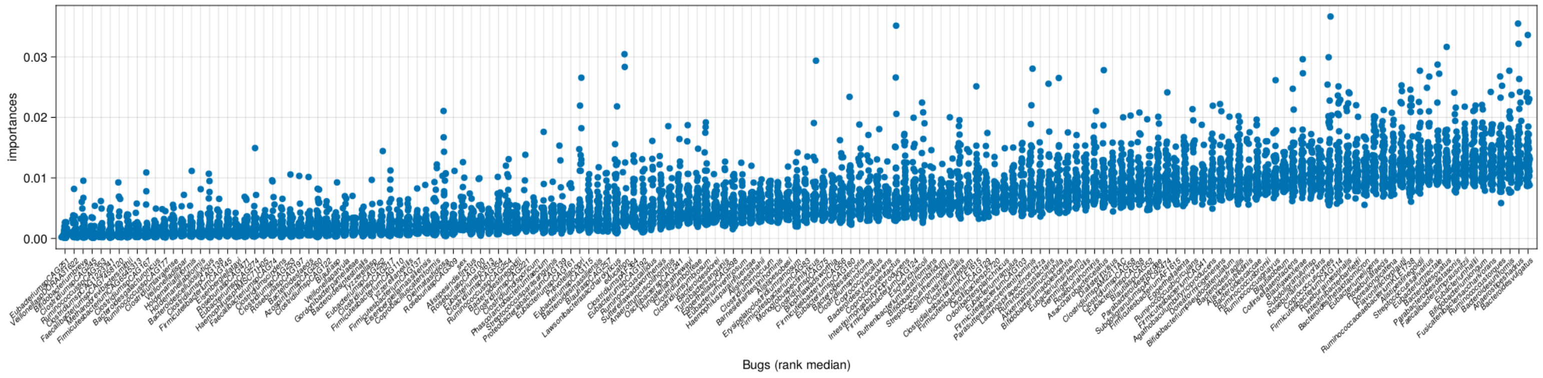

Figure S6: Median ordination of relative importances on every subcortical region shows patterns of loadings are ubiquitous irrespective of average importance. Species feature importances in brain region RFs - related to Figure 4C. All species (plus sex and maternal education) feature importances in each brain region model. X-axis is rank-ordered by median importance across all brain region models.

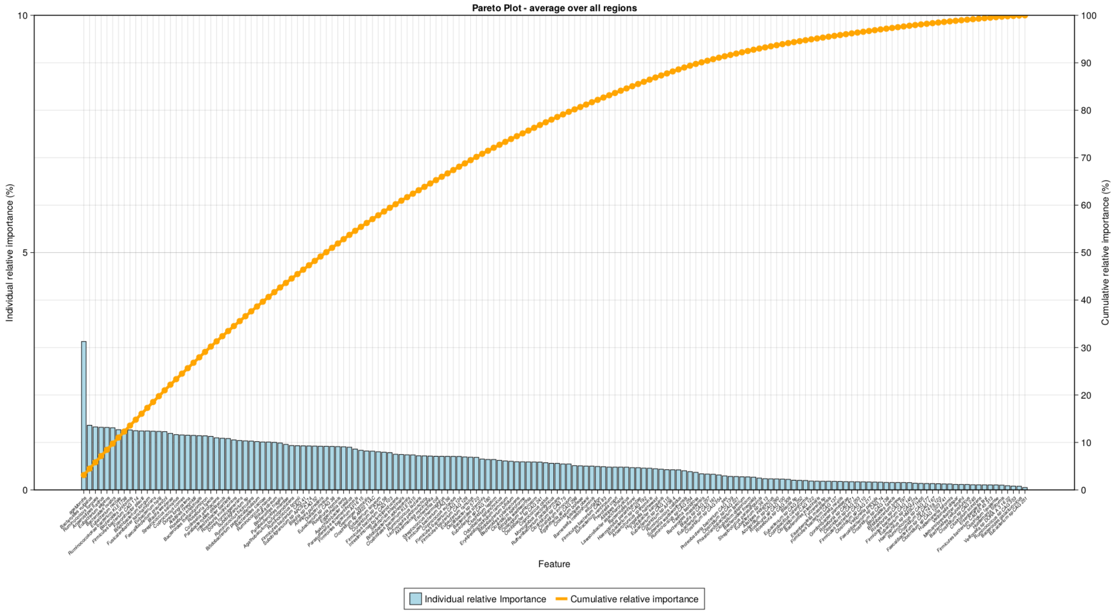

Figure S7: A small subset of species explains a large amount of the average cumulative relative importance across subcortical relative volume prediction. Pareto plots for RFs of cognitive function - related to Figure 4. The cumulative average importance (orange line), as well as ranked average importance (blue bars) of features (species) in models of brain region size.

Table S1: The top 20 important predictors account for over 30 % of cumulative importance on concurrent composite cognitive assessment score. Feature importances for RF models of cognitive performance based on taxonomic profiles in children over 18 months old.

rank	variable	weightedImportance	relativeWeightedImportance	cumulativeWeightedImportance
1	*ageMonths*	0.01907	5.99 %	5.99 %
2	*Faecalibacterium prausnitzii*	0.00701	2.2 %	8.19 %
3	*Blautia wexlerae*	0.00534	1.68 %	9.87 %
4	*Eubacterium eligens*	0.00505	1.59 %	11.45 %
5	*Bifidobacterium pseudocatenulatum*	0.00501	1.57 %	13.03 %
6	*Bifidobacterium longum*	0.00484	1.52 %	14.55 %
7	*Veillonella dispar*	0.00454	1.43 %	15.97 %
8	*Parasutterella excrementihominis*	0.00438	1.38 %	17.35 %
9	*Bacteroides vulgatus*	0.004	1.26 %	18.61 %
10	*Roseburia faecis*	0.00397	1.25 %	19.85 %
11	*Fusicatenibacter saccharivorans*	0.00385	1.21 %	21.06 %
12	*Eubacterium hallii*	0.00361	1.13 %	22.2 %
13	*Anaerostipes hadrus*	0.00358	1.12 %	23.32 %
14	*Asaccharobacter celatus*	0.00355	1.12 %	24.43 %
15	*Dorea longicatena*	0.00351	1.1 %	25.54 %
16	*Blautia obeum*	0.00347	1.09 %	26.63 %
17	*Ruminococcus gnavus*	0.00345	1.08 %	27.71 %
18	*Flavonifractor plautii*	0.00338	1.06 %	28.77 %
19	*Intestinibacter bartlettii*	0.00336	1.06 %	29.83 %
20	*Veillonella parvula*	0.00335	1.05 %	30.88 %
21	*Roseburia hominis*	0.00335	1.05 %	31.93 %
22	*Alistipes obesi*	0.00326	1.02 %	32.95 %
23	*Eubacterium sp CAG 38*	0.00323	1.01 %	33.97 %
24	*Parabacteroides distasonis*	0.00321	1.01 %	34.98 %
25	*Streptococcus parasanguinis*	0.00321	1.01 %	35.98 %
26	*Megamonas funiformis*	0.00314	0.99 %	36.97 %
27	*Bifidobacterium bifidum*	0.00309	0.97 %	37.94 %
28	*Streptococcus salivarius*	0.00303	0.95 %	38.89 %
29	*Roseburia intestinalis*	0.00303	0.95 %	39.84 %
30	*Sutterella wadsworthensis*	0.00301	0.95 %	40.79 %
31	*Eubacterium rectale*	0.00297	0.93 %	41.72 %
32	*Alistipes finegoldii*	0.00294	0.92 %	42.64 %
33	*Bacteroides uniformis*	0.0029	0.91 %	43.55 %
34	*Parabacteroides merdae*	0.00288	0.91 %	44.46 %
35	*Roseburia inulinivorans*	0.00287	0.9 %	45.36 %
36	*Firmicutes bacterium CAG 41*	0.00287	0.9 %	46.26 %
37	*Veillonella atypica*	0.00285	0.89 %	47.16 %
38	*Bacteroides ovatus*	0.00277	0.87 %	48.03 %
39	*Haemophilus parainfluenzae*	0.00277	0.87 %	48.9 %
40	*Subdoligranulum sp*	0.00276	0.87 %	49.77 %
41	*Bacteroides caccae*	0.00272	0.86 %	50.62 %
42	*Alistipes putredinis*	0.00267	0.84 %	51.46 %
43	*Monoglobus pectinilyticus*	0.00259	0.81 %	52.27 %
44	*Anaerotruncus colihominis*	0.00259	0.81 %	53.09 %
45	*Clostridium sp CAG 58*	0.00258	0.81 %	53.9 %
46	*Subdoligranulum sp APC924 74*	0.00258	0.81 %	54.71 %
47	*Collinsella aerofaciens*	0.00256	0.8 %	55.51 %
48	*Eggerthella lenta*	0.00255	0.8 %	56.31 %
49	*Bacteroides fragilis*	0.00252	0.79 %	57.1 %
50	*Ruthenibacterium lactatiformans*	0.0025	0.78 %	57.89 %
51	*Streptococcus thermophilus*	0.00246	0.77 %	58.66 %
52	*Ruminococcus torques*	0.00246	0.77 %	59.44 %
53	*Ruminococcus bromii*	0.00235	0.74 %	60.17 %
54	*Clostridium symbiosum*	0.00232	0.73 %	60.9 %
55	*Agathobaculum butyriciproducens*	0.00231	0.73 %	61.63 %
56	*Firmicutes bacterium AF16 15*	0.00227	0.71 %	62.34 %
57	*Ruminococcus bicirculans*	0.00225	0.71 %	63.05 %
58	*Prevotella copri*	0.00224	0.7 %	63.75 %
59	*Bacteroides thetaiotaomicron*	0.00223	0.7 %	64.45 %
60	*Bifidobacterium adolescentis*	0.00223	0.7 %	65.15 %
61	*Ruminococcus lactaris*	0.00219	0.69 %	65.84 %
62	*Eubacterium ramulus*	0.00215	0.68 %	66.51 %
63	*Odoribacter splanchnicus*	0.00214	0.67 %	67.19 %
64	*Dorea formicigenerans*	0.00213	0.67 %	67.85 %
65	*Eubacterium siraeum*	0.00211	0.66 %	68.52 %
66	*Erysipelatoclostridium ramosum*	0.00208	0.65 %	69.17 %
67	*Ruminococcaceae bacterium KLE1738*	0.00207	0.65 %	69.82 %
68	*Sellimonas intestinalis*	0.00205	0.64 %	70.46 %
69	*Clostridium sp AM22 11AC*	0.00201	0.63 %	71.1 %
70	*Firmicutes bacterium CAG 114*	0.00193	0.61 %	71.7 %
71	*Clostridium leptum*	0.00191	0.6 %	72.31 %
72	*Oscillibacter sp 57 20*	0.00187	0.59 %	72.89 %
73	*Eubacterium sp CAG 180*	0.00187	0.59 %	73.48 %
74	*Gemmiger formicilis*	0.00182	0.57 %	74.05 %
75	*Bacteroides xylanisolvens*	0.00181	0.57 %	74.62 %
76	*Blautia sp CAG 52*	0.00176	0.55 %	75.17 %
77	*Bacteroides dorei*	0.00175	0.55 %	75.72 %
78	*Hungatella hathewayi*	0.00173	0.54 %	76.27 %
79	*Intestinimonas butyriciproducens*	0.00172	0.54 %	76.81 %
80	*Akkermansia muciniphila*	0.00172	0.54 %	77.35 %
81	*Clostridium innocuum*	0.0017	0.54 %	77.88 %
82	*Clostridium spiroforme*	0.00169	0.53 %	78.41 %
83	*Clostridium bolteae*	0.00168	0.53 %	78.94 %
84	*Coprococcus comes*	0.00164	0.52 %	79.46 %
85	*Bifidobacterium breve*	0.00161	0.51 %	79.96 %
86	*Escherichia coli*	0.00159	0.5 %	80.46 %
87	*Eggerthella sp CAG 298*	0.00155	0.49 %	80.95 %
88	*Dialister invisus*	0.00155	0.49 %	81.44 %
89	*Clostridium sp CAG 75*	0.00154	0.48 %	81.92 %
90	*Coprococcus eutactus*	0.00154	0.48 %	82.4 %
91	*Bacteroides stercoris*	0.00152	0.48 %	82.88 %
92	*Tyzzerella nexilis*	0.00152	0.48 %	83.36 %
93	*Lachnospira pectinoschiza*	0.00151	0.47 %	83.83 %
94	*Clostridium citroniae*	0.00144	0.45 %	84.28 %
95	*Eubacterium sp 36 13*	0.00142	0.45 %	84.73 %
96	*Bilophila wadsworthia*	0.00142	0.45 %	85.18 %
97	*Firmicutes bacterium CAG 56*	0.0014	0.44 %	85.62 %
98	*Ruminococcus sp CAG 353*	0.00139	0.44 %	86.05 %
99	*Firmicutes bacterium CAG 129*	0.00138	0.43 %	86.49 %
100	*Eisenbergiella massiliensis*	0.00131	0.41 %	86.9 %
101	*Barnesiella intestinihominis*	0.0013	0.41 %	87.3 %
102	*Clostridium sp AF36 4*	0.0013	0.41 %	87.71 %
103	*Clostridiales bacterium KLE1615*	0.00123	0.39 %	88.1 %
104	…		…	…

Table S2: The top 20 important predictors account for over 30% of cumulative importance on the concurrent expressive language component of the MSEL assessment score. Feature importances for RF models of MSEL expressive language based on taxonomic profiles in children over 18 months old.

rank	variable	weightedImportance	relativeWeightedImportance	cumulativeWeightedImportance
1	*Bifidobacterium pseudocatenulatum*	0.0082	3.55 %	3.55 %
2	*ageMonths*	0.00765	3.31 %	6.87 %
3	*Blautia wexlerae*	0.0061	2.64 %	9.51 %
4	*Eubacterium eligens*	0.00502	2.18 %	11.68 %
5	*Faecalibacterium prausnitzii*	0.00499	2.16 %	13.85 %
6	*Bifidobacterium longum*	0.00402	1.74 %	15.59 %
7	*Ruminococcus gnavus*	0.00352	1.52 %	17.11 %
8	*Fusicatenibacter saccharivorans*	0.00324	1.41 %	18.52 %
9	*Roseburia inulinivorans*	0.0031	1.34 %	19.86 %
10	*Flavonifractor plautii*	0.00309	1.34 %	21.2 %
11	*Intestinibacter bartlettii*	0.00302	1.31 %	22.51 %
12	*Anaerostipes hadrus*	0.00293	1.27 %	23.78 %
13	*Streptococcus salivarius*	0.00291	1.26 %	25.03 %
14	*Parasutterella excrementihominis*	0.00286	1.24 %	26.27 %
15	*Veillonella parvula*	0.00283	1.23 %	27.5 %
16	*Bilophila wadsworthia*	0.0027	1.17 %	28.67 %
17	*Eggerthella lenta*	0.00269	1.17 %	29.84 %
18	*Veillonella dispar*	0.00258	1.12 %	30.95 %
19	*Roseburia intestinalis*	0.00256	1.11 %	32.06 %
20	*Eubacterium rectale*	0.00255	1.1 %	33.16 %
21	*Bacteroides uniformis*	0.00253	1.09 %	34.26 %
22	*Bacteroides fragilis*	0.00252	1.09 %	35.35 %
23	*Streptococcus thermophilus*	0.00251	1.09 %	36.44 %
24	*Bacteroides vulgatus*	0.00249	1.08 %	37.52 %
25	*Roseburia faecis*	0.00248	1.08 %	38.59 %
26	*Blautia sp CAG 52*	0.00244	1.06 %	39.65 %
27	*Parabacteroides distasonis*	0.00239	1.03 %	40.68 %
28	*Ruminococcus bromii*	0.00232	1.0 %	41.69 %
29	*Roseburia hominis*	0.00231	1.0 %	42.69 %
30	*Clostridium sp CAG 58*	0.0023	1.0 %	43.69 %
31	*Intestinimonas butyriciproducens*	0.00224	0.97 %	44.66 %
32	*Bacteroides ovatus*	0.00216	0.93 %	45.59 %
33	*Veillonella atypica*	0.00213	0.92 %	46.51 %
34	*Erysipelatoclostridium ramosum*	0.00212	0.92 %	47.43 %
35	*Alistipes shahii*	0.00212	0.92 %	48.35 %
36	*Barnesiella intestinihominis*	0.00209	0.91 %	49.26 %
37	*Clostridium symbiosum*	0.00208	0.9 %	50.16 %
38	*Ruthenibacterium lactatiformans*	0.00207	0.9 %	51.05 %
39	*Collinsella aerofaciens*	0.00201	0.87 %	51.92 %
40	*Firmicutes bacterium CAG 41*	0.002	0.87 %	52.79 %
41	*Bacteroides xylanisolvens*	0.00194	0.84 %	53.63 %
42	*Hungatella hathewayi*	0.00194	0.84 %	54.47 %
43	*Firmicutes bacterium AF16 15*	0.00191	0.83 %	55.29 %
44	*Anaerotruncus colihominis*	0.0019	0.82 %	56.12 %
45	*Clostridium bolteae*	0.00189	0.82 %	56.94 %
46	*Haemophilus parainfluenzae*	0.00185	0.8 %	57.74 %
47	*Bacteroides caccae*	0.00184	0.8 %	58.54 %
48	*Alistipes putredinis*	0.00181	0.79 %	59.32 %
49	*Agathobaculum butyriciproducens*	0.00181	0.78 %	60.11 %
50	*Streptococcus parasanguinis*	0.00179	0.78 %	60.88 %
51	*Blautia obeum*	0.00176	0.76 %	61.64 %
52	*Eubacterium hallii*	0.00175	0.76 %	62.4 %
53	*Parabacteroides merdae*	0.00175	0.76 %	63.16 %
54	*Sutterella wadsworthensis*	0.00174	0.76 %	63.92 %
55	*Eubacterium sp CAG 38*	0.00173	0.75 %	64.67 %
56	*Alistipes finegoldii*	0.0017	0.74 %	65.4 %
57	*Escherichia coli*	0.00169	0.73 %	66.14 %
58	*Sellimonas intestinalis*	0.00168	0.73 %	66.86 %
59	*Bacteroides thetaiotaomicron*	0.00167	0.72 %	67.59 %
60	*Ruminococcus torques*	0.00166	0.72 %	68.3 %
61	*Ruminococcus lactaris*	0.00162	0.7 %	69.01 %
62	*Ruminococcaceae bacterium KLE1738*	0.00159	0.69 %	69.7 %
63	*Firmicutes bacterium CAG 114*	0.00158	0.68 %	70.38 %
64	*Clostridium innocuum*	0.00156	0.68 %	71.06 %
65	*Clostridium clostridioforme*	0.00156	0.68 %	71.73 %
66	*Megamonas funiformis*	0.00155	0.67 %	72.4 %
67	*Asaccharobacter celatus*	0.00152	0.66 %	73.06 %
68	*Subdoligranulum sp*	0.00151	0.66 %	73.72 %
69	*Dialister invisus*	0.0015	0.65 %	74.37 %
70	*Clostridium sp AM22 11AC*	0.00148	0.64 %	75.01 %
71	*Dorea longicatena*	0.00147	0.64 %	75.65 %
72	*Bifidobacterium bifidum*	0.00147	0.64 %	76.29 %
73	*Lachnospira pectinoschiza*	0.0014	0.61 %	76.89 %
74	*Ruminococcus sp CAG 254*	0.00139	0.6 %	77.5 %
75	*Dorea formicigenerans*	0.00138	0.6 %	78.09 %
76	*Blautia sp CAG 257*	0.00137	0.6 %	78.69 %
77	*Bifidobacterium breve*	0.00133	0.58 %	79.27 %
78	*Eubacterium siraeum*	0.00133	0.58 %	79.84 %
79	*Akkermansia muciniphila*	0.0013	0.56 %	80.41 %
80	*Bifidobacterium adolescentis*	0.0013	0.56 %	80.97 %
81	*Anaerostipes caccae*	0.00124	0.54 %	81.51 %
82	*Clostridium sp CAG 122*	0.00124	0.54 %	82.04 %
83	*Clostridium sp CAG 75*	0.00122	0.53 %	82.57 %
84	*Eubacterium sp CAG 180*	0.00122	0.53 %	83.1 %
85	*Firmicutes bacterium CAG 56*	0.00121	0.52 %	83.62 %
86	*Ruminococcus bicirculans*	0.00119	0.52 %	84.14 %
87	*Odoribacter splanchnicus*	0.00112	0.49 %	84.62 %
88	*Clostridium lavalense*	0.00108	0.47 %	85.09 %
89	*Clostridium sp CAG 12237 41*	0.00107	0.46 %	85.56 %
90	*Alistipes obesi*	0.00107	0.46 %	86.02 %
91	*Clostridium spiroforme*	0.00103	0.45 %	86.46 %
92	*Bacteroides stercoris*	0.00096	0.42 %	86.88 %
93	*Tyzzerella nexilis*	0.00094	0.41 %	87.29 %
94	*Clostridium leptum*	0.00094	0.41 %	87.69 %
95	*Eggerthella sp CAG 298*	0.00091	0.39 %	88.09 %
96	*Eisenbergiella massiliensis*	0.00088	0.38 %	88.47 %
97	*Subdoligranulum sp APC924 74*	0.00085	0.37 %	88.84 %
98	*Clostridium citroniae*	0.00085	0.37 %	89.21 %
99	*Bacteroides dorei*	0.00084	0.37 %	89.57 %
100	*Eubacterium sp CAG 252*	0.00082	0.36 %	89.93 %
101	*Turicibacter sanguinis*	0.00079	0.34 %	90.27 %
102	*Gemmiger formicilis*	0.00078	0.34 %	90.61 %
103	*Phascolarctobacterium faecium*	0.00076	0.33 %	90.94 %
104	…	…	…	…

Table S3: The top 20 important predictors account for over 30% of cumulative importance on the concurrent gross motor component of the MSEL assessment score. Feature importances for RF models of MSEL gross motor based on taxonomic profiles in children over 18 months old.

rank	variable	weightedImportance	relativeWeightedImportance	cumulativeWeightedImportance
1	*ageMonths*	0.00679	4.58 %	4.58 %
2	*Roseburia faecis*	0.00358	2.41 %	6.99 %
3	*Blautia wexlerae*	0.00277	1.87 %	8.85 %
4	*Streptococcus salivarius*	0.00274	1.85 %	10.7 %
5	*Faecalibacterium prausnitzii*	0.00264	1.78 %	12.48 %
6	*Fusicatenibacter saccharivorans*	0.00249	1.68 %	14.15 %
7	*Clostridium symbiosum*	0.00238	1.6 %	15.76 %
8	*Bifidobacterium longum*	0.00231	1.55 %	17.31 %
9	*Subdoligranulum sp*	0.00221	1.49 %	18.8 %
10	*Ruminococcus gnavus*	0.00219	1.47 %	20.27 %
11	*Bifidobacterium pseudocatenulatum*	0.00218	1.47 %	21.74 %
12	*Ruminococcus bromii*	0.00218	1.47 %	23.21 %
13	*Firmicutes bacterium CAG 41*	0.00216	1.45 %	24.66 %
14	*Bacteroides uniformis*	0.00216	1.45 %	26.12 %
15	*Eubacterium sp CAG 252*	0.00207	1.39 %	27.51 %
16	*Intestinibacter bartlettii*	0.00199	1.34 %	28.85 %
17	*Anaerostipes hadrus*	0.00195	1.31 %	30.16 %
18	*Flavonifractor plautii*	0.00194	1.31 %	31.47 %
19	*Clostridiales bacterium KLE1615*	0.00189	1.28 %	32.75 %
20	*Parabacteroides distasonis*	0.00189	1.27 %	34.02 %
21	*Ruminococcus torques*	0.00178	1.2 %	35.22 %
22	*Collinsella aerofaciens*	0.00177	1.19 %	36.41 %
23	*Eubacterium sp CAG 38*	0.00173	1.17 %	37.58 %
24	*Roseburia intestinalis*	0.0017	1.15 %	38.73 %
25	*Erysipelatoclostridium ramosum*	0.00167	1.13 %	39.86 %
26	*Coprococcus catus*	0.00167	1.12 %	40.98 %
27	*Bacteroides fragilis*	0.00164	1.11 %	42.09 %
28	*Clostridium innocuum*	0.00161	1.09 %	43.17 %
29	*Clostridium sp CAG 75*	0.00161	1.08 %	44.26 %
30	*Eubacterium eligens*	0.00157	1.06 %	45.31 %
31	*Ruminococcus bicirculans*	0.00156	1.05 %	46.37 %
32	*Dorea formicigenerans*	0.00156	1.05 %	47.42 %
33	*Escherichia coli*	0.00153	1.03 %	48.45 %
34	*Bacteroides thetaiotaomicron*	0.00152	1.03 %	49.48 %
35	*Eggerthella lenta*	0.00152	1.02 %	50.5 %
36	*Sellimonas intestinalis*	0.00151	1.01 %	51.51 %
37	*Alistipes putredinis*	0.00147	0.99 %	52.51 %
38	*Bacteroides ovatus*	0.00147	0.99 %	53.5 %
39	*Eubacterium rectale*	0.00146	0.99 %	54.49 %
40	*Bacteroides vulgatus*	0.00145	0.98 %	55.47 %
41	*Roseburia inulinivorans*	0.0014	0.94 %	56.41 %
42	*Parasutterella excrementihominis*	0.0014	0.94 %	57.35 %
43	*Agathobaculum butyriciproducens*	0.00137	0.92 %	58.28 %
44	*Clostridium bolteae*	0.00135	0.91 %	59.19 %
45	*Ruminococcus lactaris*	0.00132	0.89 %	60.08 %
46	*Firmicutes bacterium CAG 56*	0.0013	0.88 %	60.96 %
47	*Ruthenibacterium lactatiformans*	0.0013	0.88 %	61.83 %
48	*Eubacterium hallii*	0.00129	0.87 %	62.7 %
49	*Coprococcus comes*	0.00122	0.82 %	63.53 %
50	*Bifidobacterium bifidum*	0.00121	0.81 %	64.34 %
51	*Bilophila wadsworthia*	0.00119	0.8 %	65.15 %
52	*Hungatella hathewayi*	0.00116	0.78 %	65.93 %
53	*Haemophilus parainfluenzae*	0.00114	0.77 %	66.7 %
54	*Streptococcus thermophilus*	0.00114	0.77 %	67.47 %
55	*Bacteroides caccae*	0.00111	0.75 %	68.21 %
56	*Clostridium sp AM22 11AC*	0.0011	0.74 %	68.96 %
57	*Roseburia hominis*	0.0011	0.74 %	69.69 %
58	*Blautia obeum*	0.00108	0.73 %	70.42 %
59	*Ruminococcus sp CAG 254*	0.00105	0.71 %	71.13 %
60	*Firmicutes bacterium AF16 15*	0.00103	0.7 %	71.83 %
61	*Alistipes finegoldii*	0.001	0.67 %	72.5 %
62	*Megamonas funiformis*	0.00089	0.6 %	73.1 %
63	*Intestinimonas butyriciproducens*	0.00089	0.6 %	73.7 %
64	*Blautia sp CAG 257*	0.00086	0.58 %	74.27 %
65	*Anaerotruncus colihominis*	0.00084	0.57 %	74.84 %
66	*Gemmiger formicilis*	0.00084	0.57 %	75.41 %
67	*Dielma fastidiosa*	0.00083	0.56 %	75.97 %
68	*Subdoligranulum sp APC924 74*	0.00083	0.56 %	76.53 %
69	*Dorea longicatena*	0.00082	0.55 %	77.08 %
70	*Bifidobacterium adolescentis*	0.00082	0.55 %	77.63 %
71	*Clostridium sp CAG 58*	0.00081	0.55 %	78.18 %
72	*Clostridium lavalense*	0.00079	0.53 %	78.71 %
73	*Clostridium leptum*	0.00078	0.53 %	79.24 %
74	*Bacteroides stercoris*	0.00078	0.53 %	79.77 %
75	*Clostridium sp CAG 122*	0.00078	0.53 %	80.29 %
76	*Eubacterium ramulus*	0.00078	0.52 %	80.82 %
77	*Holdemania filiformis*	0.00077	0.52 %	81.34 %
78	*Eubacterium sp 36 13*	0.00077	0.52 %	81.86 %
79	*Ruminococcaceae bacterium KLE1738*	0.00077	0.52 %	82.38 %
80	*Firmicutes bacterium CAG 114*	0.00076	0.52 %	82.89 %
81	*Clostridium sp CAG 12237 41*	0.00076	0.51 %	83.41 %
82	*Lachnospira pectinoschiza*	0.00075	0.51 %	83.91 %
83	*Parabacteroides merdae*	0.00072	0.49 %	84.4 %
84	*Dialister invisus*	0.00071	0.48 %	84.88 %
85	*Eisenbergiella massiliensis*	0.00068	0.46 %	85.34 %
86	*Streptococcus parasanguinis*	0.00067	0.45 %	85.79 %
87	*Turicibacter sanguinis*	0.00067	0.45 %	86.24 %
88	*Akkermansia muciniphila*	0.00066	0.44 %	86.68 %
89	*Tyzzerella nexilis*	0.00064	0.43 %	87.11 %
90	*Clostridium aldenense*	0.00062	0.42 %	87.53 %
91	*Bacteroides dorei*	0.00062	0.41 %	87.95 %
92	*Clostridium spiroforme*	0.0006	0.4 %	88.35 %
93	*Phascolarctobacterium faecium*	0.00058	0.39 %	88.74 %
94	*Bacteroides xylanisolvens*	0.00058	0.39 %	89.13 %
95	*Bifidobacterium breve*	0.00057	0.39 %	89.52 %
96	*Veillonella dispar*	0.00055	0.37 %	89.89 %
97	*Eubacterium siraeum*	0.00054	0.36 %	90.25 %
98	*Asaccharobacter celatus*	0.00053	0.36 %	90.61 %
99	*Clostridium sp CAG 354*	0.00053	0.36 %	90.97 %
100	*Prevotella copri*	0.00048	0.32 %	91.29 %
101	*Coprococcus eutactus*	0.00047	0.32 %	91.61 %
102	*Odoribacter splanchnicus*	0.00045	0.31 %	91.92 %
103	*Oscillibacter sp 57 20*	0.00044	0.3 %	92.21 %
104	…	…	…	…

Table S4: : The top 20 important predictors account for over 30% of cumulative importance on the concurrent visual reception component of the MSEL assessment score. Feature importances for RF models of MSEL visual reception based on taxonomic profiles in children over 18 months old.

rank	variable	weightedImportance	relativeWeightedImportance	cumulativeWeightedImportance
1	*ageMonths*	0.01081	4.69 %	4.69 %
2	*Faecalibacterium prausnitzii*	0.00534	2.32 %	7.01 %
3	*Bifidobacterium pseudocatenulatum*	0.00394	1.71 %	8.72 %
4	*Roseburia faecis*	0.00391	1.7 %	10.42 %
5	*Clostridium innocuum*	0.00385	1.67 %	12.09 %
6	*Bacteroides vulgatus*	0.00379	1.64 %	13.73 %
7	*Blautia wexlerae*	0.00374	1.62 %	15.35 %
8	*Bifidobacterium longum*	0.00371	1.61 %	16.96 %
9	*Parabacteroides merdae*	0.00334	1.45 %	18.41 %
10	*Eubacterium sp CAG 38*	0.00329	1.43 %	19.84 %
11	*Intestinibacter bartlettii*	0.00314	1.36 %	21.2 %
12	*Anaerostipes hadrus*	0.00299	1.3 %	22.5 %
13	*Megamonas funiformis*	0.00286	1.24 %	23.74 %
14	*Eggerthella lenta*	0.0027	1.17 %	24.91 %
15	*Eubacterium eligens*	0.00265	1.15 %	26.06 %
16	*Ruminococcus bromii*	0.00261	1.13 %	27.2 %
17	*Ruminococcus gnavus*	0.00261	1.13 %	28.33 %
18	*Parasutterella excrementihominis*	0.0026	1.13 %	29.46 %
19	*Streptococcus salivarius*	0.0026	1.13 %	30.59 %
20	*Bacteroides uniformis*	0.00246	1.07 %	31.65 %
21	*Roseburia inulinivorans*	0.00246	1.07 %	32.72 %
22	*Dorea longicatena*	0.00244	1.06 %	33.78 %
23	*Bacteroides dorei*	0.00242	1.05 %	34.83 %
24	*Flavonifractor plautii*	0.00239	1.04 %	35.87 %
25	*Fusicatenibacter saccharivorans*	0.00235	1.02 %	36.89 %
26	*Intestinimonas butyriciproducens*	0.0023	1.0 %	37.89 %
27	*Roseburia intestinalis*	0.00229	0.99 %	38.88 %
28	*Eubacterium hallii*	0.00228	0.99 %	39.87 %
29	*Streptococcus thermophilus*	0.00227	0.98 %	40.85 %
30	*Veillonella dispar*	0.00225	0.97 %	41.82 %
31	*Bacteroides caccae*	0.00223	0.97 %	42.79 %
32	*Haemophilus parainfluenzae*	0.00223	0.97 %	43.76 %
33	*Roseburia hominis*	0.00223	0.97 %	44.73 %
34	*Alistipes finegoldii*	0.0022	0.96 %	45.68 %
35	*Eubacterium rectale*	0.0022	0.96 %	46.64 %
36	*Collinsella aerofaciens*	0.00212	0.92 %	47.56 %
37	*Parabacteroides distasonis*	0.00209	0.91 %	48.46 %
38	*Bacteroides thetaiotaomicron*	0.00204	0.88 %	49.35 %
39	*Sellimonas intestinalis*	0.00203	0.88 %	50.23 %
40	*Ruthenibacterium lactatiformans*	0.00202	0.88 %	51.11 %
41	*Ruminococcus bicirculans*	0.00202	0.88 %	51.98 %
42	*Blautia obeum*	0.002	0.87 %	52.85 %
43	*Firmicutes bacterium CAG 41*	0.00198	0.86 %	53.71 %
44	*Ruminococcus lactaris*	0.00198	0.86 %	54.57 %
45	*Bacteroides ovatus*	0.0019	0.83 %	55.4 %
46	*Hungatella hathewayi*	0.00189	0.82 %	56.22 %
47	*Anaerotruncus colihominis*	0.00188	0.82 %	57.03 %
48	*Bacteroides fragilis*	0.00186	0.81 %	57.84 %
49	*Erysipelatoclostridium ramosum*	0.00186	0.81 %	58.65 %
50	*Veillonella atypica*	0.00182	0.79 %	59.44 %
51	*Subdoligranulum sp*	0.00181	0.79 %	60.22 %
52	*Ruminococcus torques*	0.0018	0.78 %	61.0 %
53	*Clostridium symbiosum*	0.00175	0.76 %	61.77 %
54	*Bifidobacterium bifidum*	0.00172	0.75 %	62.51 %
55	*Escherichia coli*	0.00172	0.74 %	63.26 %
56	*Veillonella parvula*	0.00172	0.74 %	64.0 %
57	*Sutterella wadsworthensis*	0.00171	0.74 %	64.74 %
58	*Clostridium sp CAG 58*	0.00168	0.73 %	65.47 %
59	*Dorea formicigenerans*	0.00167	0.73 %	66.2 %
60	*Bacteroides xylanisolvens*	0.00166	0.72 %	66.92 %
61	*Prevotella copri*	0.00163	0.71 %	67.62 %
62	*Ruminococcaceae bacterium KLE1738*	0.00163	0.71 %	68.33 %
63	*Monoglobus pectinilyticus*	0.0016	0.69 %	69.02 %
64	*Agathobaculum butyriciproducens*	0.00159	0.69 %	69.71 %
65	*Akkermansia muciniphila*	0.00155	0.67 %	70.39 %
66	*Firmicutes bacterium AF16 15*	0.00154	0.67 %	71.06 %
67	*Clostridium bolteae*	0.00154	0.67 %	71.72 %
68	*Streptococcus parasanguinis*	0.00147	0.64 %	72.36 %
69	*Eubacterium siraeum*	0.00144	0.63 %	72.99 %
70	*Coprobacillus cateniformis*	0.00143	0.62 %	73.61 %
71	*Lachnospira pectinoschiza*	0.00143	0.62 %	74.23 %
72	*Clostridium sp AF36 4*	0.00143	0.62 %	74.85 %
73	*Firmicutes bacterium CAG 114*	0.00141	0.61 %	75.47 %
74	*Blautia sp CAG 52*	0.00139	0.6 %	76.07 %
75	*Bifidobacterium breve*	0.00136	0.59 %	76.66 %
76	*Clostridium sp AM22 11AC*	0.00133	0.58 %	77.24 %
77	*Clostridium leptum*	0.00133	0.58 %	77.82 %
78	*Bilophila wadsworthia*	0.00131	0.57 %	78.38 %
79	*Bifidobacterium adolescentis*	0.00129	0.56 %	78.94 %
80	*Barnesiella intestinihominis*	0.00128	0.56 %	79.5 %
81	*Alistipes putredinis*	0.00127	0.55 %	80.05 %
82	*Alistipes obesi*	0.00124	0.54 %	80.59 %
83	*Subdoligranulum sp APC924 74*	0.00117	0.51 %	81.09 %
84	*Coprococcus comes*	0.00113	0.49 %	81.58 %
85	*Tyzzerella nexilis*	0.00113	0.49 %	82.07 %
86	*Eubacterium sp CAG 252*	0.00104	0.45 %	82.52 %
87	*Dialister invisus*	0.00102	0.44 %	82.96 %
88	*Gemmiger formicilis*	0.00101	0.44 %	83.4 %
89	*Asaccharobacter celatus*	0.00098	0.43 %	83.83 %
90	*Turicibacter sanguinis*	0.00098	0.42 %	84.25 %
91	*Clostridium spiroforme*	0.00097	0.42 %	84.68 %
92	*Blautia sp CAG 257*	0.00097	0.42 %	85.09 %
93	*Clostridium sp CAG 122*	0.00097	0.42 %	85.51 %
94	*Eggerthella sp CAG 298*	0.00096	0.42 %	85.93 %
95	*Lactococcus lactis*	0.00095	0.41 %	86.34 %
96	*Bacteroides stercoris*	0.00093	0.4 %	86.74 %
97	*Clostridium clostridioforme*	0.00092	0.4 %	87.14 %
98	*Anaerostipes caccae*	0.00091	0.39 %	87.53 %
99	*Eisenbergiella massiliensis*	0.0009	0.39 %	87.93 %
100	*Clostridium sp CAG 75*	0.00088	0.38 %	88.31 %
101	*Firmicutes bacterium CAG 65 45 313*	0.00088	0.38 %	88.69 %
102	*Clostridium lavalense*	0.00085	0.37 %	89.06 %
103	*Clostridium aldenense*	0.00084	0.36 %	89.42 %
104	…	…	…	…

Table S5: The top 20 important predictors account for over 50% of cumulative importance on future composite cognitive assessment score. Feature importances for RF models of future cognitive performance based on taxonomic profiles in children over 18 months old.

rank	variable	weightedImportance	relativeWeightedImportance	cumulativeWeightedImportance
1	*future ageMonths*	0.00289	3.5 %	3.5 %
2	*Klebsiella pneumoniae*	0.00274	3.31 %	6.81 %
3	*Klebsiella variicola*	0.00251	3.03 %	9.85 %
4	*Bacteroides ovatus*	0.00248	3.0 %	12.84 %
5	*Intestinibacter bartlettii*	0.00238	2.88 %	15.72 %
6	*Escherichia coli*	0.00237	2.86 %	18.58 %
7	*Clostridium innocuum*	0.00235	2.84 %	21.42 %
8	*present ageMonths*	0.00218	2.63 %	24.05 %
9	*Ruminococcus gnavus*	0.00216	2.61 %	26.66 %
10	*Bacteroides vulgatus*	0.00212	2.57 %	29.23 %
11	*Veillonella dispar*	0.00205	2.47 %	31.7 %
12	*Clostridium neonatale*	0.00202	2.45 %	34.15 %
13	*Bifidobacterium longum*	0.00202	2.44 %	36.59 %
14	*Veillonella parvula*	0.00198	2.4 %	38.99 %
15	*Flavonifractor plautii*	0.00175	2.11 %	41.1 %
16	*Erysipelatoclostridium ramosum*	0.00162	1.95 %	43.05 %
17	*Veillonella atypica*	0.00161	1.95 %	45.0 %
18	*Bifidobacterium breve*	0.00159	1.93 %	46.93 %
19	*Streptococcus parasanguinis*	0.00149	1.8 %	48.73 %
20	*Eggerthella lenta*	0.00148	1.79 %	50.52 %
21	*Streptococcus salivarius*	0.00142	1.71 %	52.24 %
22	*Klebsiella oxytoca*	0.00137	1.65 %	53.89 %
23	*Clostridioides difficile*	0.00136	1.65 %	55.54 %
24	*Klebsiella quasipneumoniae*	0.00134	1.62 %	57.16 %
25	*Parabacteroides distasonis*	0.00133	1.61 %	58.76 %
26	*Haemophilus parainfluenzae*	0.00124	1.5 %	60.27 %
27	*Bifidobacterium bifidum*	0.00121	1.47 %	61.73 %
28	*Enterococcus avium*	0.00118	1.42 %	63.16 %
29	*Hungatella hathewayi*	0.00115	1.39 %	64.55 %
30	*Lactobacillus rhamnosus*	0.00111	1.34 %	65.89 %
31	*Enterococcus faecalis*	0.0011	1.33 %	67.22 %
32	*Bacteroides uniformis*	0.00109	1.32 %	68.54 %
33	*Collinsella aerofaciens*	0.00101	1.22 %	69.76 %
34	*Enterobacter cloacae complex*	0.001	1.21 %	70.97 %
35	*Tyzzerella nexilis*	0.00088	1.07 %	72.04 %
36	*Bacteroides dorei*	0.00088	1.07 %	73.1 %
37	*Clostridium paraputrificum*	0.00082	1.0 %	74.1 %
38	*Roseburia faecis*	0.00081	0.98 %	75.08 %
39	*Faecalibacterium prausnitzii*	0.00077	0.93 %	76.01 %
40	*Haemophilus sp HMSC71H05*	0.00077	0.93 %	76.94 %
41	*Veillonella infantium*	0.00076	0.92 %	77.86 %
42	*Klebsiella michiganensis*	0.00074	0.9 %	78.76 %
43	*Anaerostipes caccae*	0.00074	0.9 %	79.65 %
44	*Bacteroides fragilis*	0.00074	0.9 %	80.55 %
45	*Bifidobacterium pseudocatenulatum*	0.00069	0.84 %	81.39 %
46	*Bifidobacterium dentium*	0.00068	0.82 %	82.21 %
47	*Bilophila wadsworthia*	0.00068	0.82 %	83.03 %
48	*Bacteroides thetaiotaomicron*	0.00065	0.79 %	83.82 %
49	*Bacteroides caccae*	0.00062	0.75 %	84.57 %
50	*Roseburia intestinalis*	0.0006	0.72 %	85.29 %
51	*Bifidobacterium adolescentis*	0.00058	0.71 %	86.0 %
52	*Parabacteroides merdae*	0.00057	0.69 %	86.69 %
53	*Megasphaera micronuciformis*	0.00053	0.65 %	87.33 %
54	*Eubacterium sp CAG 38*	0.00052	0.63 %	87.96 %
55	*Enterococcus gallinarum*	0.0005	0.61 %	88.57 %
56	*Fusicatenibacter saccharivorans*	0.00049	0.59 %	89.16 %
57	*Sutterella wadsworthensis*	0.00047	0.57 %	89.72 %
58	*Anaerostipes hadrus*	0.00042	0.51 %	90.23 %
59	*Clostridium aldenense*	0.00041	0.5 %	90.73 %
60	*Bacteroides stercoris*	0.00041	0.5 %	91.23 %
61	*Blautia wexlerae*	0.00041	0.5 %	91.73 %
62	*Sellimonas intestinalis*	0.00041	0.5 %	92.22 %
63	*Ruminococcus torques*	0.00041	0.49 %	92.71 %
64	*Ruthenibacterium lactatiformans*	0.0004	0.48 %	93.19 %
65	*Clostridium sp 7 2 43FAA*	0.0004	0.48 %	93.67 %
66	*Parasutterella excrementihominis*	0.00037	0.44 %	94.12 %
67	*Akkermansia muciniphila*	0.00035	0.42 %	94.54 %
68	*Clostridium symbiosum*	0.00034	0.41 %	94.95 %
69	*Clostridium bolteae*	0.00033	0.4 %	95.35 %
70	*Streptococcus mitis*	0.00032	0.39 %	95.74 %
71	*Citrobacter freundii*	0.00029	0.35 %	96.1 %
72	*Eubacterium rectale*	0.00028	0.34 %	96.44 %
73	*Firmicutes bacterium CAG 41*	0.00028	0.34 %	96.78 %
74	*Veillonella sp DORA A 3 16 22*	0.00028	0.33 %	97.12 %
75	*Megasphaera sp MJR8396C*	0.00023	0.28 %	97.4 %
76	*Roseburia inulinivorans*	0.00022	0.27 %	97.66 %
77	*Firmicutes bacterium CAG 424*	0.00021	0.26 %	97.92 %
78	*Clostridium perfringens*	0.00021	0.26 %	98.18 %
79	*Blautia sp CAG 257*	0.00019	0.23 %	98.41 %
80	*Anaerotruncus colihominis*	0.00018	0.22 %	98.63 %
81	*Prevotella copri*	0.00016	0.19 %	98.83 %
82	*Alistipes finegoldii*	0.00016	0.19 %	99.01 %
83	*Clostridium clostridioforme*	0.00016	0.19 %	99.2 %
84	*Eubacterium hallii*	0.00015	0.18 %	99.38 %
85	*Streptococcus thermophilus*	0.00015	0.18 %	99.56 %
86	*Blautia obeum*	0.00015	0.18 %	99.74 %
87	*Bacteroides xylanisolvens*	0.00014	0.16 %	99.91 %
88	*Dorea longicatena*	8.0e-5	0.09 %	100.0 %

Table S6: Breakdown of validation-set benchmarks show differential predictive power of gut microbiome along several subcortical brain regions. RF model performances predicting cortical and subcortical brain regions.

Segment	Mean absolute proportional error (MAPE)	Correlation coefficient (R)
Left orbitofrontal	0.0554	0.2121
Right orbitofrontal	0.0601	0.2002
Left anterior cingulate	0.0684	0.0619
Right anterior cingulate	0.0737	0.2185
Left lateral occipital	0.062	0.2313
Right lateral occipital	0.0547	0.199
Left thalamus proper	0.0523	0.1823
Right thalamus proper	0.049	0.1955
Left pallidum	0.0641	0.2707
Right pallidum	0.0611	0.2476
Left amygdala	0.0634	0.2271
Right amygdala	0.0707	0.1701
Left accumbens area	0.0951	0.2745
Right accumbens area	0.1036	0.005
Left cuneus	0.0795	0.2899
Right cuneus	0.074	0.1353
Left entorhinal	0.0788	0.1677
Right entorhinal	0.0824	0.0323
Left fusiform	0.0573	0.2998
Right fusiform	0.0534	0.2125
Left lingual	0.0671	0.4437
Right lingual	0.071	0.4408
Left parahippocampal	0.0568	0.1925
Right parahippocampal	0.0609	0.114
Left paracentral	0.0752	0.2601
Right paracentral	0.0822	0.1112
Left pericalcarine	0.1102	0.2674
Right pericalcarine	0.0895	0.3153
Left precentral	0.0647	0.2485
Right precentral	0.0463	0.0771
Left precuneus	0.0568	0.129
Right precuneus	0.0689	0.1709

Table S7: The experimental design and input composition for Random Forest experiments included multiple data types and combinations

<table>
<tr><th>Input set</th><th>Age bracket</th><th>Microbiome encoding type</th><th>Demographics Provided? (sex, education)</th></tr>
<tr><td>1</td><td rowspan="5">0 to 6 months</td><td>Not provided</td><td>yes</td></tr>
<tr><td>2</td><td rowspan="2">Taxonomic profile</td><td>no</td></tr>
<tr><td>3</td><td>yes</td></tr>
<tr><td>4</td><td rowspan="2">Functional Profile (ECs)</td><td>no</td></tr>
<tr><td>5</td><td>yes</td></tr>
<tr><td>6</td><td rowspan="5">18 to 120 months</td><td>Not provided</td><td>yes</td></tr>
<tr><td>7</td><td rowspan="2">Taxonomic profile</td><td>no</td></tr>
<tr><td>8</td><td>yes</td></tr>
<tr><td>9</td><td rowspan="2">Functional Profile (ECs)</td><td>no</td></tr>
<tr><td>10</td><td>yes</td></tr>
</table>
